# R-spondin 3 promotes stem cell recovery and epithelial regeneration in the colon

**DOI:** 10.1038/s41467-019-12349-5

**Published:** 2019-09-25

**Authors:** Christine Harnack, Hilmar Berger, Agne Antanaviciute, Ramon Vidal, Sascha Sauer, Alison Simmons, Thomas F. Meyer, Michael Sigal

**Affiliations:** 1 Medical Department, Division of Gastroenterology and Hepatology, Charité University Medicine, 13353 Berlin, Germany; 2 Department of Molecular Biology, Max Planck Institute for Infection Biology, 10117 Berlin, Germany; 3 Nuffield Department of Medicine and MRC Human Immunology Unit, University of Oxford, Oxford, OX3 7BN UK; 4 Max Delbrück Center, 13125 Berlin, Germany; 5 Berlin Institute of Health, 10117 Berlin, Germany

**Keywords:** Cell biology, Intestinal stem cells, Gastroenterology

## Abstract

The colonic epithelial turnover is driven by crypt-base stem cells that express the R-spondin receptor Lgr5. Signals that regulate epithelial regeneration upon stem cell injury are largely unknown. Here, we explore the dynamics of Wnt signaling in the colon. We identify two populations of cells with active Wnt signaling: highly proliferative Lgr5^+^/Axin2^+^ cells, as well as secretory Lgr5^−^/Axin2^+^ cells. Upon Lgr5^+^ cell depletion, these cells are recruited to contribute to crypt regeneration. Chemical injury induced by DSS leads to a loss of both Lgr5^+^ cells and Axin2^+^ cells and epithelial regeneration is driven by Axin2^−^ cells, including differentiated Krt20^+^ surface enterocytes. Regeneration requires stromal Rspo3, which is present at increased levels upon injury and reprograms Lgr5^−^ but Lgr4^+^ differentiated cells. In contrast, depletion of stromal Rspo3 impairs crypt regeneration, even upon mild injury. We demonstrate that Rspo3 is essential for epithelial repair via induction of Wnt signaling in differentiated cells.

## Abstract (web summary)

Epithelial turnover in the colon requires stem cells in the crypt that express the R-spondin receptor Lgr5. Here, the authors show that regeneration after colon injury involving loss of Lgr5^+^ and Axin2^+^ cells requires stromal derived Rspo3-dependent reprogramming of Lgr4^+^ differentiated cells, including Krt20^+^ enterocytes.

## Introduction

The colon epithelium is characterized by high turnover kinetics. This constant replacement of the epithelium is driven by cells located in the base of colonic crypts, which constantly divide and give rise to the various differentiated cell types. During this process, the cells migrate along the crypt wall towards the surface where they are shed into the lumen. The majority of epithelial cells are therefore short-lived, in contrast to a limited number of long-lived stem cells in the base of the crypts. Lgr5 is a well-established marker of these basal stem cells in the gastrointestinal tract^1^ and lineage tracing experiments have demonstrated that Lgr5^+^ cells give rise to clonal cell populations establishing entire colonic crypts^2^.

While Lgr5^+^ cells are long-lived and act as stem cells during homeostatic conditions, experimental models have demonstrated that Lgr5^+^ stem cells per se are not essential for maintaining epithelial integrity^3^. This has been studied mainly in the small intestine, where different cell types such as enterocyte progenitors^4^, more differentiated secretory Bmi1^+^ cells^3^, or even fully differentiated Paneth cells can repopulate crypts upon injury. In the colon, this process has not been investigated in detail, but recent studies demonstrated the ability of Atoh1^+^ secretory cells to partially restore depleted Lgr5^+^ cells^5,6^.

Expression of *Lgr5* is controlled by Wnt signaling, which plays a critical role for stem cell turnover in the gastrointestinal tract^2^. In contrast to the small intestine, where epithelial Paneth cells as well as stromal cells act as niche cells that express Wnt ligands, stromal cells have recently been identified as a major source of Wnt ligands and Rspo in the colon^7,8^. Rspo proteins are secreted and can stabilize the effects of Wnt ligands by preventing ubiquitination and turnover of the Wnt receptor frizzled^9^, thereby dictating the size of the Lgr5^+^ stem cell pool by regulating self-renewal of Lgr5^+^ cells^10^. We have shown that in the stomach, stem cell homeostasis is regulated by Wnt and Rspo secreted by stromal myofibroblasts^11^. More recently, Rspo3 depletion has been shown to increase sensitivity to chemical injury in the colon, but the exact mechanisms are not clear^12^.

Here, we explore the dynamics of crypt regeneration and Wnt/Rspo signaling in the colon in the context of crypt injury. We demonstrate that while Rspo3 from myofibroblasts maintains colonic Lgr5^+^ cells during homeostasis, during injury its main function is not to maintain Lgr5^+^ cells but to interact with more differentiated cells that express Lgr4 but not Lgr5 and are able to regain expression of Wnt target genes and generate new crypts. This Rspo3-driven regeneration program is supported by injury-induced stromal remodeling, and is essential for epithelial recovery. In mice lacking Rspo3, injury repair is almost completely abolished. Thus, we find that endogenous Rspo3 signaling is a critical determinant of cellular fate within the crypt and stimulates rapid recruitment of differentiated cells for epithelial wound healing and crypt regeneration through induction of Wnt signaling.

## Results

### Axin2 marks crypt base stem cells and secretory progenitors

**Fig. 1:**
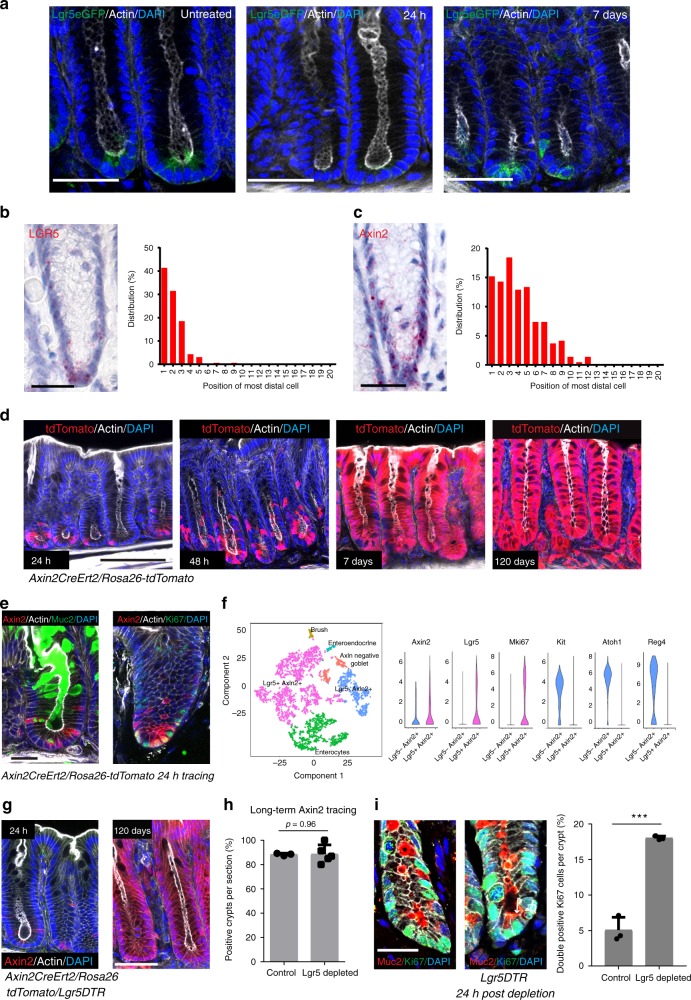
Wnt-responsive Axin2^+^ cells restore the colonic Lgr5^+^ stem cell compartment upon depletion. **a** Immunofluorescence images from the colon of *Lgr5DTReGFP* mice, left untreated (left panel) or treated with a single dose of diphtheria toxin 24 h (middle) or 7 days (right) before sacrifice (scale bar = 50 µm). **b** Single-molecule in situ hybridization for *Lgr5* and determination of *Lgr5*^+^ cell positions counted from the crypt base. **c** Single-molecule in situ hybridization for *Axin2* and determination of *Axin2*^+^ cell positions counted from the crypt base. **d** Immunofluorescence images from the colon of *Axin2CreErt2/Rosa-26tdTomato* mice, showing Axin2 lineage tracing for 24 h, 48 h, 7 days and 120 days induced by a single dose of tamoxifen. **e** Images of colon tissue from *Axin2CreErt2/Rosa-26tdTomato* mice showing *Axin2* lineage tracing for 24 h, co-stained for MUC2 (left), and Ki67 (right) (scale bar = 25 µm). **f** t-SNE plot of single-cell RNAseq data from colon crypts, violin plots for selected genes expressed in the Lgr5^+^/Axin^+^ compared to the Lgr5^−^/Axin^+^ population. **g** Immunofluorescence images of the colon of triple heterozygous *Lgr5DTReGFP/Axin2CreErt2/Rosa26-tdTomato* mice treated either with a single dose of diphtheria toxin (to ablate Lgr5^+^ cells) and tamoxifen (to start Axin2^+^ cell tracing) 24 h before sacrifice (left panel) or a single dose of tamoxifen plus three doses of diphtheria toxin on 3 consecutive days 120 days before sacrifice (right panel) (scale bar = 50 µm). **h** Quantification of tdTomato^+^ crypts in *Axin2CreErt2/Rosa26-tdTomato* animals treated with a single dose of tamoxifen (control *n* = 3 mice), and *Lgr5DTReGFP/Axin2CreErt2/Rosa26-tdTomato* mice treated with a single dose of tamoxifen plus three doses of diphtheria toxin on 3 consecutive days (Lgr5 depletion *n* = 5 mice). Animals were sacrificed after 60 days (scale bar = 50 µm). **i** Confocal images of colon tissue of control and Lgr5DTR mice treated with diphtheria toxin three times, and labeled after 24 h recovery for Muc2 (red) and Ki67 (green); quantification of double-positive cells per crypt (*n* = 3 mice per group, scale bar = 25 µm). Data represent mean ± SD; Student’s *t*-test (two-tailed) was applied in all cases. ***p < 0.001. Scale bar = 100 µm unless indicated otherwise. Source data are provided as a source data file

To study how Lgr5^+^ cell depletion affects crypt integrity in the colon, we applied the *Lgr5eGFP-DTR* mouse model, in which Lgr5^+^ cells can be selectively depleted by injection of diphtheria toxin (DT)^11^. Expression of *Lgr5* was restricted essentially to cells in positions 1–3 of the crypt (Fig. 1a), a finding that was confirmed by single molecule in situ hybridization (ISH) (Fig. 1b). DT injection resulted in depletion of the Lgr5^+^ cell compartment within 24 h, followed by recovery at day 7 post-DT injection (Fig. 1a). This finding led us to ask which signals were responsible for recovery of the Lgr5^+^ cell compartment.

Lgr5 is a Wnt target gene specifically expressed in the cells with the highest Wnt signaling^2^. We hypothesized that, similar to the stomach^11^, colon crypts might exhibit a Wnt gradient with Wnt-high cells in the very base and cells that are exposed to a lower concentration of Wnt above the basal compartment. Using ISH to examine the expression pattern of the classical Wnt target gene *Axin2*, we observed transcripts distributed more broadly than *Lgr5*, yet mostly limited to positions 1–5 of the crypt (Fig. 1c). Using *Axin2CreErt2/Rosa26-tdTomato/Lgr5eGFP-DTR* triple heterozygous mice, which express GFP under the *Lgr5* promoter, treated with tamoxifen to induce tdTomato in Axin2^+^ cells 24 h before sacrifice, we confirmed that Lgr5^+^ cells in the crypt base co-express Axin2 (Supplementary Fig. 1a). Lineage tracing using *Axin2CreErt2/Rosa26-tdTomato* mice revealed that Axin2^+^ cells populated almost every crypt, demonstrating that this cell population contained the stem cell compartment (Fig. 1d). To test whether Axin2^+^ cells contribute to crypt recovery upon Lgr5^+^ cell depletion, we used *Axin2CreErt2/Rosa26-tdTomato/Lgr5DTR* triple heterozygous mice. Lgr5^+^ cells were depleted using DT (daily on days 1–3) and Axin2-lineage tracing was initiated by tamoxifen (on day 1). We observed that upon depletion of Lgr5^+^ cells the remaining, Lgr5^−^/Axin2^+^ cells repopulated the colonic crypts, including the crypt base (Fig. 1g). Quantification of the lineage-tracing data revealed that 88% of the crypts were repopulated by the Axin2^+^ cell compartment, even upon Lgr5^+^ cell ablation, similar to the mice with a functional Lgr5^+^ cell compartment (Fig. 1h). To better define the identity of Axin2 cells, we sorted Axin2^+^ and Axin2^−^ epithelial cells from *Axin2CreErt2/Rosa26-tdTomato* mice, treated with tamoxifen 24 h before sacrifice (Supplementary Fig. 1b), and performed microarray analysis.

Gene set enrichment analysis revealed an enrichment of Lgr5^+^ stem cell signature genes, as well as genes involved in proliferation in Axin2^+^ cells (Supplementary Fig. 1d). To further dissect the heterogeneity between Lgr5^+^ and Lgr5^−^ Axin2^+^ cells, we analyzed the recently published single-cell RNAseq data set from colon crypts^13^. We observed that *Axin2* was expressed in all subpopulations of Lgr5^+^ cells, which we collectively assigned as Lgr5^+^ Axin2^+^ cell population (Fig. 1f, pink). In addition, *Axin2* was found in a cluster that did not express *Lgr5* (blue). These cells expressed less *Ki67*, but instead *Atoh1*, *c-kit*, and *Reg4*, known markers of crypt base goblet cells (Fig. 1f). Co-staining of Axin2 and Ki67, as well as Muc2 confirmed that while most Axin2^+^ cells are proliferative, a subpopulation marks crypt base goblet cells (Fig. 1e). We depleted Lgr5^+^ cells in Lgr5^−^DTR mice for 3 consecutive days and co-stained the colon for Ki67 and Muc2. Almost no double-positive cells were found in the control animals, whereas in mice with a depleted Lgr5^+^ cell compartment, we found an increased number of Muc2^+^/Ki67^+^ cells (Fig. 1i), while the overall number of Ki67 cells per crypt was not altered (Supplementary Fig. 2a, b). Overall this indicates that Axin2^+^ goblet cells de-differentiate upon Lgr5^+^ cell depletion to maintain the proliferative activity and regenerate the crypt.

### Rspo3 determines the colonic Lgr5^+^ stem cell signature

**Fig. 2:**
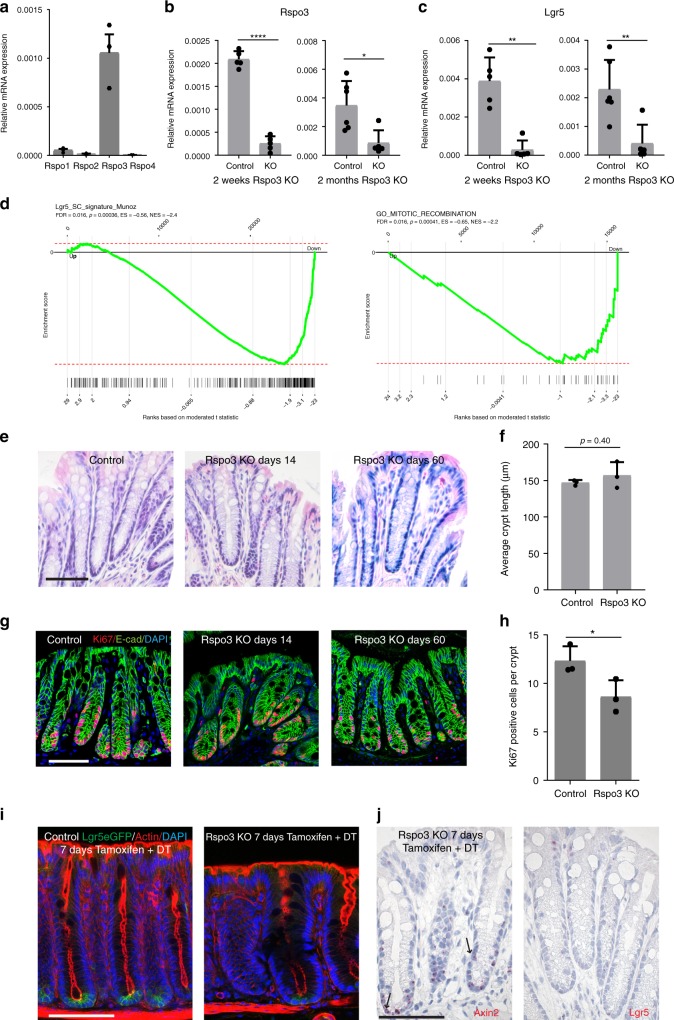
Rspo3 from in Myh11^+^ myofibroblasts determines the stem cell signature and is required for Lgr5^+^ cell recovery. **a** qPCR for *Rspo* homologs from colon tissue from *n* = 3 mice. **b** qPCR for *Rspo3* in *Myh11CreErt2/Rspo3*^*fl/fl*^ mice (*Rspo3* KO) and corresponding *Myh11CreErt2/Rspo3*^*+/+*^ control mice at 2 weeks (*n* = 5 mice per group) and 2 months (*n* = 6 control mice, *n* = 5 *Rspo3* KO mice) after injection of tamoxifen into *Rspo3* KO and corresponding controls (2 months data: Mann–Whitney *U*-test (two-tailed) was performed). **c** qPCR for Lgr5 *in Myh11CreErt2/Rspo3*^*fl/fl*^ mice (*Rspo3* KO) and corresponding *Myh11CreErt2/Rspo3*^*+/+*^ control mice at 2 weeks (*n* = 5 mice per group) and 2 months (*n* = 6 control mice, *n* = 5 *Rspo3* KO mice) after injection of tamoxifen into *Rspo3* KO and corresponding controls (Mann–Whitney *U*-test (two-tailed). **d** GSEA analysis of genes differentially expressed in colon tissue from *Rspo3* KO versus control mice in comparison to the previously published small intestinal Lgr5^+^ stem cell signature gene set and in comparison to the mitotic recombination related signature gene set obtained from MSigDB (data from two microarrays from independent biological replicates per group). **e** H&E staining of the colon from a control and an *Rspo3* KO mouse at 14 and 60 days after tamoxifen treatment, showing no marked anatomical differences. **f** Average crypt length in colon tissue from control (*n* = 3 mice) and an *Rspo3* KO (*n* = 4 mice) mice 60 days after tamoxifen treatment. **g** Confocal microscopy image of colon tissue from control and *Rspo3* KO mice at 14 and 60 days after tamoxifen treatment stained for Ki67 (red), E-cadherin (green), and DAPI. **h** Quantification of Ki67^+^ cells per crypt section of control and *Rspo3* KO mice 60 days after tamoxifen treatment (*n* = 3 mice per group). **i** Confocal microscopy images from the colon of an *Lgr5DTReGFP/Myh11CreErt2/Rspo3*^*fl/fl*^ and an *Lgr5DTReGFP/Myh11CreErt2/Rspo3*^*+/+*^ mouse treated with a single dose of tamoxifen and DT 7 days before sacrifice. **j** Single-molecule in situ hybridization for *Axin2* and *Lgr5* on colon tissue from an *Lgr5DTReGFP/Myh11CreErt2/Rspo3*^*fl/fl*^ mouse treated with DT and tamoxifen (scale bar = 100 µm). Data represent mean ± SD, Student’s *t*-test (two tailed) was applied in all cases, unless otherwise specified. *p < 0.05, **p < 0.01, ***p < 0.001, ****p < 0.0001. Scale bar = 100 µm unless indicated otherwise. Source data are provided as a source data file

Since Rspo proteins stabilize and enhance Wnt signaling and are therefore responsible for maintaining expression of *Lgr5* in gastric stem cells^11^, we explored the expression of different *Rspos* in the colon. As in the stomach, *Rspo3* showed the highest expression among the four *Rspo* family members (Fig. 2a). Single-molecule ISH confirmed *Rspo3* expression in the colon, concentrated in the stromal compartment that surrounds the crypt base (Supplementary Fig. 2c, left). Using *Myh11CreErt2/Rosa26-tdTomato* mice treated with tamoxifen to visualize the myofibroblast lineage, we found that Myh11^+^ cells are present at the same position in the lamina muscularis mucosae where Rspo3 transcripts were observed (Supplementary Fig. 2c, right). To investigate whether *Rspo3* is indeed expressed in Myh11^+^ cells, as in the stomach, we used our conditional *Myh11CreErt2/Rspo3*^*fl/fl*^ knockout mouse model (*Rspo3 KO*)^11^, which enables conditional depletion of *Rspo3* in Myh11-expressing myofibroblasts. We observed an 84% reduction of *Rspo3* expression 2 weeks post-induction, which remained stable for 2 months (Fig. 2b). Conditional depletion of *Rspo3* led to a rapid downregulation of *Lgr5* expression in the colon (Fig. 2c, Supplementary Fig. 2d). This was confirmed using ISH for *Lgr5* (Supplementary Fig. 2e). In addition, ISH for *Axin2* showed that expression was also reduced, albeit not completely (Supplementary Fig. 2e). In contrast to the colon, in the small intestine depletion of *Rspo3* in myofibroblasts did not regulate expression of *Rspo3*, *Axin2*, and *Lgr5*, indicating that additional sources of Rspo in the small intestine exist (Supplementary Fig. 5 a, b). Indeed, expression of Axin2, as a marker of active Wnt signaling, was less restricted in the small intestine compared to the colon (Supplementary Fig. 5c).

To gain a broader view of how endogenous Rspo3 affects the transcriptome in the colon, we performed microarray analysis of RNA from the colon of *Rspo3 KO* mice and corresponding wild-type controls at 2 weeks post-depletion. Gene set enrichment analysis revealed that Rspo3 depletion leads to a significant downregulation of Lgr5^+^ cell signature genes as previously defined by Munoz et al. ^14^ for the small intestine (Fig. 2d). Despite the down-regulation of Lgr5^+^ stem cell genes, *Rspo3 KO* mice did not develop notable epithelial injury or abnormalities (Fig. 2e, f), nor did they show detectable pathology or impaired digestion reflected by diarrhea or weight loss, indicating that stromal Rspo3 is not essential for colon crypt integrity, consistent with a recent report^12^. Ki67 staining, however, showed that proliferation was slightly reduced at 2 months after Rspo3 depletion (Fig. 2g, h). Accordingly, the microarray data confirmed significant down-regulation of proliferation-associated gene sets, including “Go Mitotic Recombination“ (Fig. 2d). Nonetheless, during normal homeostasis, crypt function and gross anatomy appeared to be maintained in the absence of Rspo3.

Next, we bred *Lgr5DTR/Myh11CreErt2/Rspo3*^*fl/fl*^ mice to ask whether epithelial integrity can be maintained if Lgr5^+^ cells are depleted by DT and the *Rspo3* gene is simultaneously deleted in myofibroblasts by injection of tamoxifen. Seven days later, no Lgr5^+^ cells were found in the gland base of animals that received tamoxifen and DT, while the Lgr5 cell compartment had recovered in Rspo3 proficient (*Lgr5DTR/Myh11CreErt2/Rspo3*^*+/+*^) animals that had received the same treatment (Fig. 2i). This indicates that regeneration of Lgr5^+^ cells in the gland based upon depletion requires stromal Rspo3. Notably, although regeneration of the Lgr5^+^ cells was impaired, the tissue did not appear substantially altered. We noticed that while Lgr5 expression was almost completely lost upon Rspo3 depletion, some Axin2 signal was still present (Fig. 2j), indicating that Wnt signaling can be maintained upon Rspo3 loss at levels that are sufficient to maintain crypt homeostasis.

### Rspo3 is essential for epithelial integrity during colitis

**Fig. 3:**
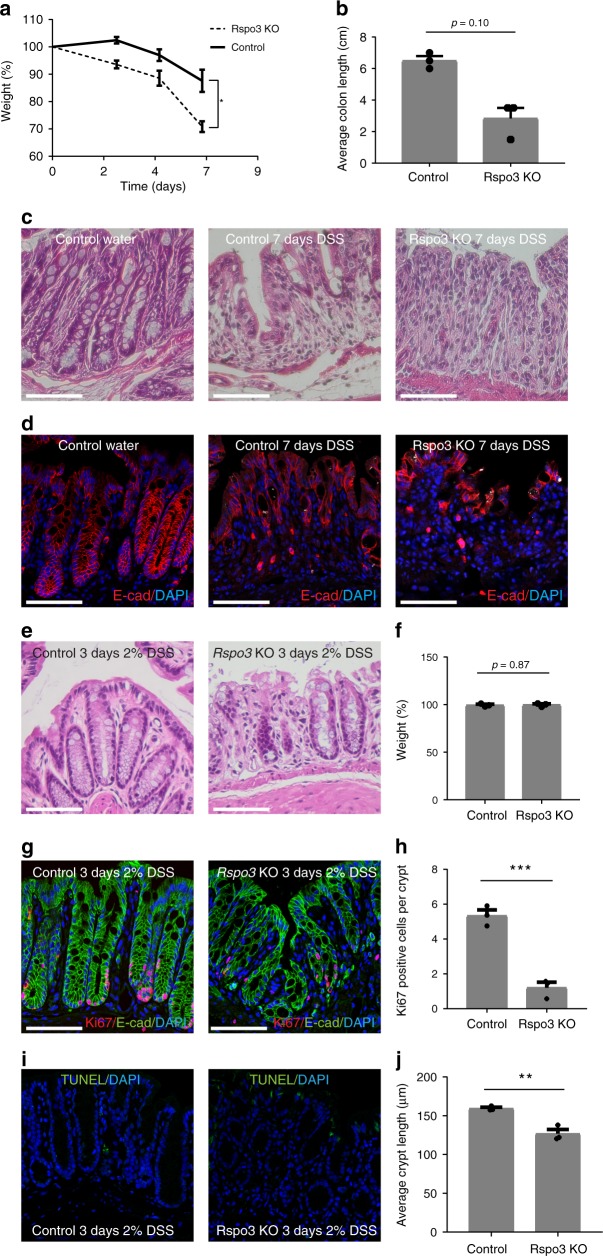
Endogenous Rspo3 is crucial during DSS-induced injury. **a** Weight curves of control and *Rspo3* KO mice during DSS treatment (*n* = 3 mice per group). **b** Comparison of colon length in control and *Rspo3* KO mice sacrificed on day 7 of DSS treatment (*n* = 3 mice per group (*p* = 0.1000, Mann–Whitney *U*-test (two-tailed)). **c** H&E staining of colon tissue from an untreated control mouse, a DSS-treated control mouse and a DSS-treated *Rspo3* KO mouse sacrificed at 7 days of DSS treatment. The *Rspo3* KO mouse shows an almost complete loss of the epithelial lining. **d** Confocal microscopy of the colon epithelium detected by immunofluorescence labeling of E-cadherin in an untreated control mouse, a DSS-treated control mouse, and a DSS-treated *Rspo3* KO mouse sacrificed at 7 days of DSS treatment. **e** H&E staining of colon tissue from a control and an *Rspo3* KO mouse treated with DSS for 3 days. **f** Weight of WT and *Rspo3* KO mice on day 3 of DSS treatment, *n* = 3 mice per group. **g** Confocal microscopy images displaying Ki67 expression in the colon of a control and *Rspo3* KO mouse. **h** Quantification of Ki67^+^ cells per crypt section in control and *Rspo3* KO mice treated with DSS for 3 days (*n* = 3 mice per group). **i** Confocal microscopy images displaying apoptosis in the colon of a control and *Rspo3* KO mouse on day 3 of DSS treatment. **j** Average crypt length in control and *Rspo3* KO mice treated with DSS for 3 days (*n* = 3 mice per group). All experiments were performed in at least three biological replicates. Data represent mean ± SD, Student’s *t*-test (two-tailed) was applied in all cases, unless otherwise specified. *p < 0.05, **p < 0.01, ***p < 0.001, ****p < 0.0001. Scale bar = 100 µm unless indicated otherwise. Source data are provided as a source data file

Loss of stromal Rspo3 in the colon was recently shown to exacerbate colitis induced by DSS, a well-established chemical colitogen that causes damage of the colon epithelium^15^. To test whether this applies to our conditional knockout model, we treated our *Myh11CreErt2/Rspo3*^*fl/fl*^ (*Rspo3* KO) and *Myh11CreErt2/Rspo3*^*+/+*^ mice with DSS after depleting Rspo3 by tamoxifen injection. Within 7 days of DSS treatment, all *Rspo3* KO mice either died or developed severe weight loss of >20%, requiring euthanasia, whereas control animals experienced only mild weight loss (Fig. 3a). At dissection, the *Rspo3* KO mice already exhibited major macroscopic alterations in the colon with a 50% reduction in length compared to the *Myh11CreErt2/Rspo3*^*+/+*^ control group (Fig. 3b). Histological analysis revealed that in *Rspo3* KO animals the epithelial lining of the colon was almost completely lost, with no detectable crypt architecture, while in the wild-type controls the injury was much less pronounced and a crypt-like epithelial architecture was retained (Fig. 3c). Immunofluorescence staining for E-cadherin confirmed an almost complete loss of the epithelium in the *Rspo3* KO animals (Fig. 3d). We repeated the experiment with 2% instead of 2.5% DSS but observed a similar phenotype in the *Rspo3* KO animals, with severe weight loss (Supplementary Fig. 3a) and epithelial injury (Supplementary Fig. 3b, c).

To obtain insight into the function of Rspo3 in the context of injury we repeated the DSS experiment but sacrificed the animals earlier (day 3), before significant weight loss occurred (Fig. 3f). On day 3, the crypt architecture in the *Myh11CreErt2/Rspo3*^*+/+*^ controls appeared intact while the crypt epithelium of *Rspo3* KO animals showed severe damage, with a complete loss of crypt bases (Fig. 3e) accompanied by an almost complete loss of the proliferative compartment (Fig. 3g, h, j), indicating that Rspo3 prevents the loss of the proliferative cells in the gland base. Of note, we did not observe a substantial number of apoptotic cells upon DSS treatment in the crypt base compartment (Fig. 3i), indicating that the crypt loss is a result of impaired proliferation and self-renewal, rather than increased apoptosis in the crypt base.

### DSS-driven injury leads to a loss of stem cells

**Fig. 4:**
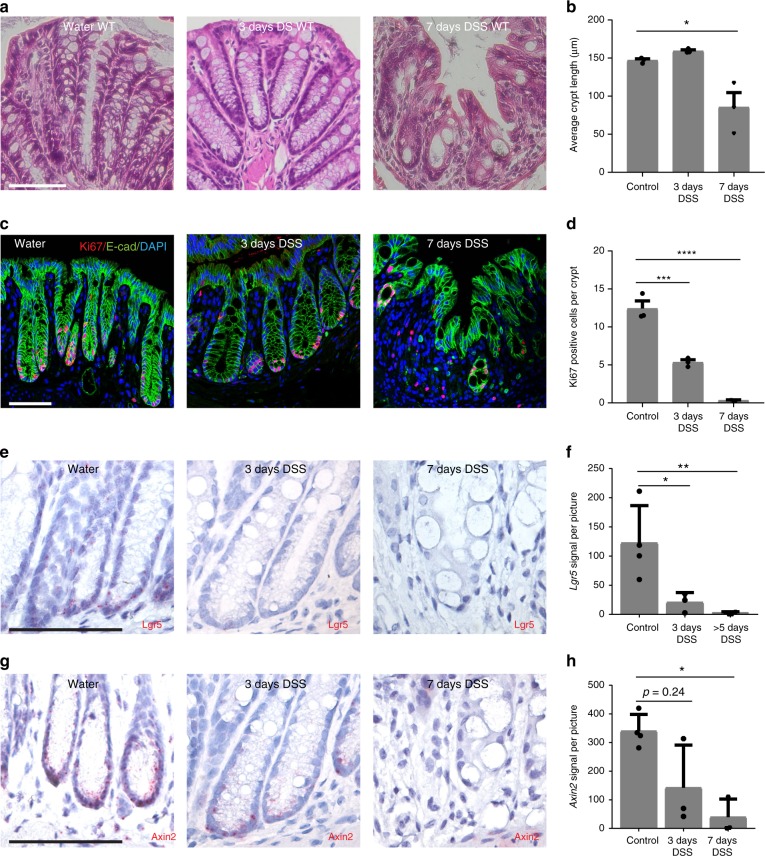
DSS-induced loss of the crypt bases containing stem and reserve stem cells. **a** H&E staining of colon tissue from untreated mice (left) and mice treated with DSS for 3 days (middle) and 7 days (right). **b** Average crypt length of control, 3 days DSS treated and 7 days DSS-treated animals (*n* = 3 mice per group). **c** Immunofluorescence labeling for Ki67 in tissue from untreated mice (left) and mice treated with DSS for 3 days (middle) and 7 days (right). **d** Quantification of Ki67-positive cells per crypt (data from *n* = 3 mice per group). **e** Single-molecule in situ hybridization for *Lgr5* in tissue from untreated mice (left) and mice treated with DSS for 3 days (middle) and 7 days (right). **f** Quantification of Lgr5 signal per image of tissue shown in e (images from *n* = 3 to *n* = 4 mice per group). **g** Single-molecule in situ hybridization for *Axin2* in tissue from untreated mice (left) and mice treated with DSS for 3 days (middle) and 7 days (right). **h** Quantification of Axin2 signal per image shown in **g** (images from *n* = 3 to *n* = 4 mice per group, Kruskal–Wallis test with Dunn's post hoc test was used). All experiments were performed in at least three biological replicates. Data represent mean ± SD; one-way ANOVA and Dunnett´s test for correction of multiple testing was applied in all cases, unless otherwise specified. *p < 0.05, **p < 0.01, ***p < 0.001, ****p < 0.0001. Scale bar = 100 µm unless indicated otherwise. Source data are provided as a source data file

Since Rspo3 is a major determinant of Lgr5^+^ stem cell identity and essential for maintaining the proliferative compartment upon DSS colitis, we asked how DSS affects the stem cell pool. The fact that the tissue appeared intact on day 3 of DSS treatment in wild-type mice (Fig. 4a, b), a time when proliferating cells were still present in the base (Fig. 4c, d), suggested that the ability to withstand DSS injury could be mediated by an expansion of the proliferative Lgr5^+^ cells in the presence of Rspo3. To visualize the Lgr5^+^ cell compartment, we thus performed single-cell ISH for *Lgr5* in DSS-treated wild-type mice. We found that the Lgr5 compartment was almost completely lost by day 3, even though the crypts still appeared relatively intact (Fig. 4e, f). Next, we asked how the compartment of reserve Axin2^+^ stem cells responds to DSS and observed a reduced expression of Axin2^+^ on day 3 and an almost complete loss on day 7 of DSS treatment (Fig. 4g, h). Therefore, Rspo3 maintains proliferation even in the absence of Lgr5^+^ stem cells or Lgr5^−^/Axin2^+^ cells.

### Axin2^**−**^ differentiated cells contribute to crypt regeneration

**Fig. 5:**
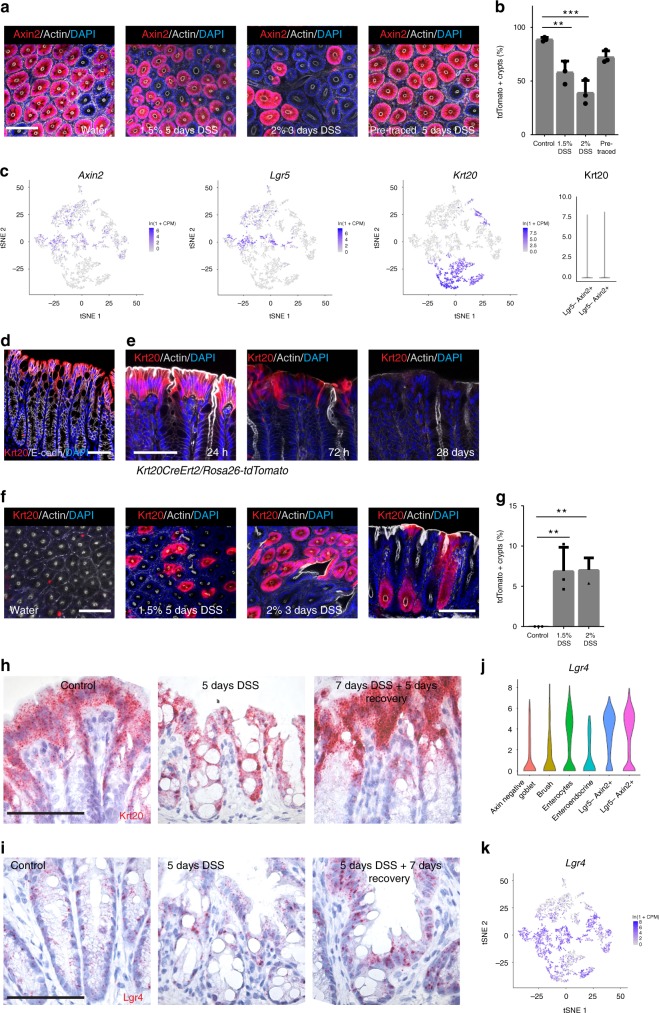
Differentiated epithelial cells are reprogrammed to act as stem cells during recovery. **a** Confocal microscopy images from whole mounts of the colon from *Axin2CreErt2/Rosa26-tdTomato* mice either untreated or treated with 1.5% or 2% DSS. A single dose of tamoxifen was injected into the animals on the last day of DSS treatment except the pre-traced mouse, where lineage tracing was performed for 1 month before DSS treatment. Lineage tracing and recovery was carried out for 1 month (scale bar = 100 µm). **b** Quantification of tdTomato-positive crypts from animals shown in **a** (control *n* = 4 mice, DSS-treated *n* = 3 mice, pre-traced *n* = 3 mice). **c** t-SNE plots of RNA single-cell sequencing show no overlap of the Lgr5^+^ or Axin2^+^ cell clusters with the Krt20^+^ cell population; violin plots for Krt20 and Lgr4 in these two populations. **d** Immunofluorescence labeling for Krt20 and E-Cadherin in tissue from untreated mice (scale bar = 100 µm). **e** Confocal microscopy images of colon tissue from *Krt20CreErt2/Rosa-26tdTomato* mice, showing Krt20 lineage tracing for 24 h, 27 h, and 28 days induced by a single dose of tamoxifen (scale bar = 50 µm). **f** Confocal microscopy images from whole mounts of the colon from *Krt20CreErt2/Rosa26-tdTomato* mice either untreated or treated with 1.5% or 2% DSS. A single dose of tamoxifen was injected into the animals on the last day of DSS treatment. Lineage tracing and recovery was carried out for 1 month (scale bar = 100 µm). **g** Quantification of tdTomato-positive crypts from animals shown in **f** (*n* = 3 mice per group). **h** Single-molecule ISH for *Krt20* and **i** single-molecule ISH for *Lgr4* in colon tissue of untreated control mice (column 1), mice sacrificed at day 5 of DSS treatment (column 2) and mice treated with DSS and sacrificed after another 5 days of recovery (column 3) (scale bar = 100 µm, *n* = 3 mice per group). **j** Violin plot for Lgr4 expression in colon epithelial subpopulation. **k** t-SNE plot of RNA single-cell sequencing for Lgr4 shows expression in all populations. Data represent mean ± SD, one-way ANOVA, and Dunnett´s test for correction of multiple testing was applied in all cases, *p < 0.05, **p < 0.01, ***p < 0.001, ****p < 0.0001. Scale bar = 100 µm unless indicated otherwise. Source data are provided as a source data file

Our data indicated that Rspo3-driven maintenance of the crypt integrity could be due to an interaction with more differentiated cells. Thus, we asked which cells are responsible for crypt regeneration. *Axin2CreErt2/Rosa26-tdTomato* mice were treated with either 2% DSS for 3 days or 1.5% DSS for 5 days and injected with tamoxifen on the last day of DSS treatment. Mice were then allowed to recover for 1 month before sacrifice (Fig. 5a). While in untreated animals the vast majority (90%) of crypts were labeled, in mice treated with 1.5% or 2% DSS, 40% and 60% of crypts were tdTomato negative, respectively (Fig. 5b), indicating that a high proportion of crypts were generated from Axin2^−^ cells upon injury. To address whether the Axin2^−^ progeny of Axin2^+^ cells is responsible for crypt regeneration, we induced Axin2 lineage tracing for one month to allow the crypts to become tdTomato positive and then treated animals with DSS for 5 days, followed by one month of recovery. We found that a high proportion of regenerated crypts remained tdTomato positive (Fig. 5a, last panel). Together, these data indicate that differentiated Axin2^−^ cells can recover the epithelium upon injury.

Since the Axin2^+^ cell pool was lost upon DSS injury, we asked which cells are able to maintain epithelial integrity and crypt recovery. Using our microarray data from Axin2^+^ versus Axin2^−^ cells, we identified *Krt20* as a subset marker of Axin2^−^ cells. Analyzing colonic single-cell RNAseq data, we confirmed that *Krt20* is exclusively expressed in the Axin2^−^ enterocyte lineage (Fig. 5c) and we confirmed its restricted expression at the surface of the crypt using immunofluorescence (Fig. 5d) and RNA ISH (Fig. 5h). We also visualized the gene expression of other genes that have recently been used for lineage tracing in the context of DSS. *Notch1*^+^ cells, which did not contribute to crypt regeneration upon DSS^6^ showed a similar expression pattern as *Lgr5*^+^ cells (Supplementary Fig. 1c), which are lost upon DSS treatment. *Krt19*, which did contribute to crypt regeneration^6^ was in fact highly expressed in all cell types including stem cells and all differentiated cell types.

We therefore focused on Krt20^+^ cells. Immunofluorescence confirmed that Krt20 was expressed specifically in surface enterocytes (Fig. 5d). To test whether enterocytes are re-programmed upon injury, we bred *Krt20CreErt2* to *Rosa-tdTomato* reporter mice. 24 h after injection of tamoxifen, we observed expression of *tdTomato* specifically in surface enterocytes. Lineage tracing for 4 weeks resulted in complete loss of marked epithelial cells in the colon (Fig. 5e). In contrast, if mice were treated with DSS for 3 or 5 days and lineage tracing induced on the last day of treatment, we found marked crypts at 4 weeks after initiation of lineage tracing, demonstrating that even the most differentiated surface enterocytes are recruited to the stem cell pool upon injury (Fig. 5f, g). We confirmed the expression pattern of *Krt20* using ISH and observed that Krt20^+^ cells were found in the tissue during DSS treatment and upon recovery (Fig. 5h). We asked how Krt20^+^ cells could interact with Rspo3, since they no longer express its receptor Lgr5. Single-cell RNAseq data and ISH showed that surface enterocytes, as well as all other differentiated cells, expressed high levels of the Rspo receptor gene *Lgr4* (Fig. 5k, j), even in mice treated with DSS (Fig. 5i)—indicating that they retain the ability to interact with Rspo and to reactivate Wnt signaling.

### Reprogrammed differentiated cells drive crypt regeneration

**Fig. 6:**
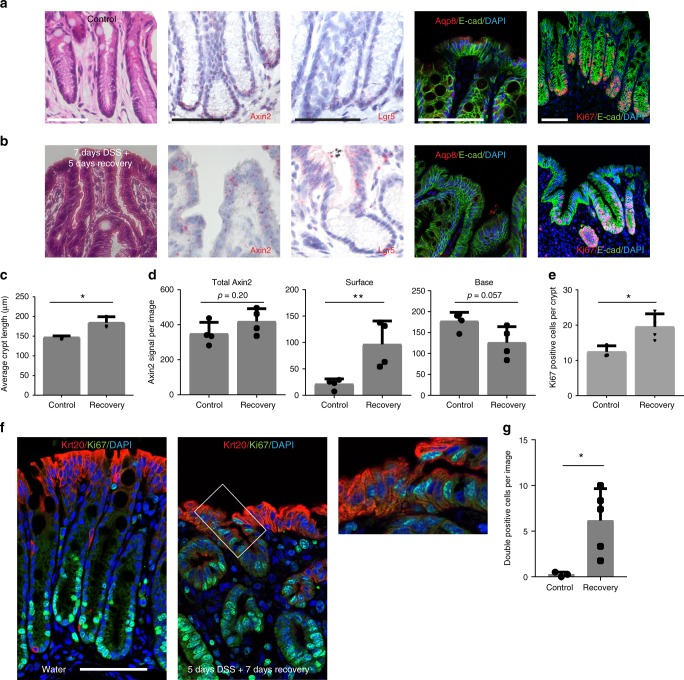
Re-expression of Wnt signaling in surface enterocytes drives epithelial recovery. **a, b** H&E staining (column 1), single-molecule ISH for Axin2 (column 2) and Lgr5 (column 3) and confocal microscopy images showing aquaporin 8 (column 4) and Ki67 (column 5) protein expression in the colon tissue of untreated control mice **a**, and mice treated with DSS for 7 days and sacrificed after another 5 days of recovery **b**. **c** Average crypt length in colon tissue in control and recovery animals (*n* = 3 per group). **d** Quantification of Axin2 signal per image in total, at the surface of crypts and in the base of crypts (*n* = 4 mice per group). **e** Quantification of Ki67^+^ cells per crypt (*n* = 3 mice in control group, *n* = 4 mice in recovery group). **f** Confocal microscopy images of Ki67 and Krt20 co-staining in untreated mice and mice after 5 days DSS treatment. **g** Quantification of double-positive cells per image (*n* = 3 mice per group). All experiments were performed in at least three biological replicates. Data represent mean ± SD; Student’s *t*-test (two-tailed) was applied in all cases. *p < 0.05, **p < 0.01, ***p < 0.001, ****p < 0.0001. Scale bar = 100 µm unless indicated otherwise. Source data are provided as a source data file

To gain further insight into the processes that regulate epithelial regeneration, we treated animals with DSS for 7 days and euthanized them after 5 days of recovery. Analysis of tissue from untreated (Fig. 6a) and 5 days recovered mice (Fig. 6b) showed that on day 5 of recovery the animals had re-gained their proliferative compartment. Quantification of Ki67 staining revealed that there is an increased proliferation at the stage of epithelial recovery (Fig. 6e), as well as increased length of crypts (Fig. 6c). Interestingly, proliferation was not limited to the crypt base but was present even in the surface cells (Fig. 6b right panel), while in untreated animals proliferating cells were restricted to the crypt (Fig. 6a right panel). ISH revealed a strong expression of both *Axin2* and *Lgr5* in the epithelium (Fig. 6b, d), again not limited to the crypt but also present in the surface epithelium (Fig. 6b, d). In contrast, expression of the surface enterocyte marker aquaporin 8 (Aqp8), which has recently been shown to be induced in colon organoids upon withdrawal of Wnt^16^, was decreased during recovery (Fig. 6b, fourth panel).

Since we found that Krt20^+^ cells give rise to crypts upon injury (Fig. 5f, g) we performed co-labeling for Krt20 and Ki67. Almost no co-labeled cells were found in untreated animals but at 7 days after DSS treatment double-positive cells were present, confirming de-differentiation of Krt20^+^ enterocytes in the context of epithelial injury repair (Fig. 6f, g).

### Rspo3^+^ cells are indispensable for epithelial recovery

**Fig. 7:**
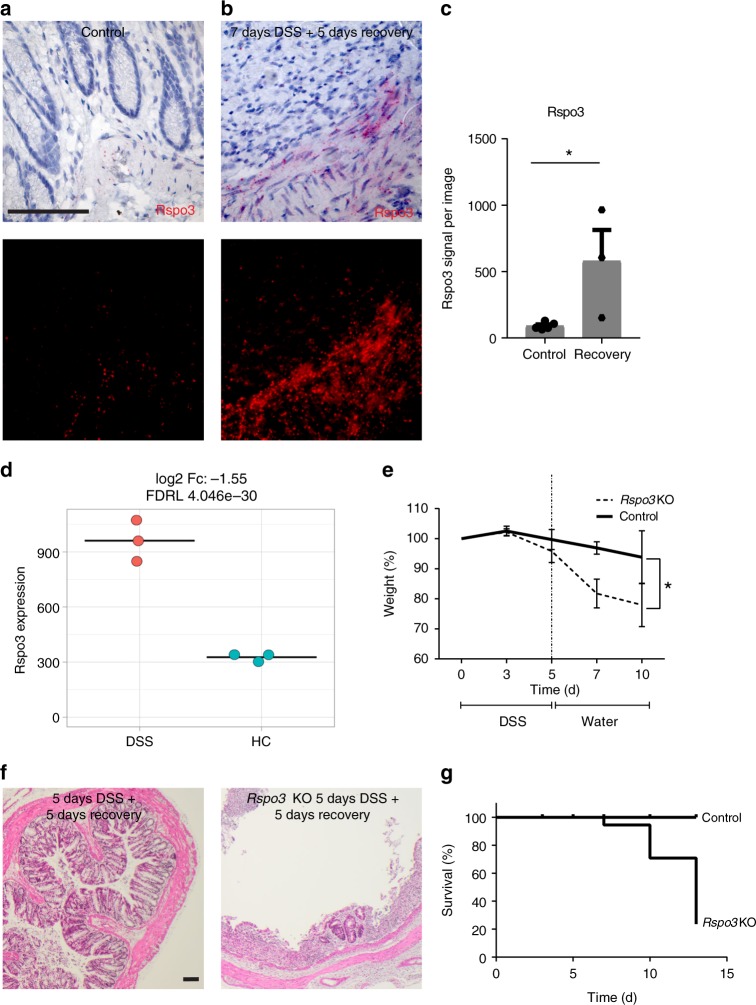
Stromal Rspo3 expression is increased upon DSS treatment. **a** single-molecule ISH for *Rspo3* in tissue from control animals (row 1) and the corresponding fluorescence image, scale bar = 100 µm. **b** Single-molecule ISH for *Rspo3* in tissue from 7 days DSS-treated animals on day 5 of recovery (row 1) and the corresponding fluorescence image. **c** Quantification of Rspo3 signal from images of **a** and **b** (*n* = 5 mice from the control and *n* = 3 mice from the DSS group). **d** Pseudo-bulk expression of Rspo3 in the stroma cells from mice either treated with DSS or healthy controls, *n* = 3 mice per group analyzed. **e** Weight curves of 5 days 1.5% DSS-treated control (*n* = 5 mice) and *Rspo3* KO (*n* = 6 mice) mice demonstrating weight loss after DSS was discontinued. **f** H&E staining of control animals and *Rspo3* KO animals on day 5 of recovery after 5 days of DSS treatment. **g** Kaplan–Meier curve of control (*n* = 5 mice) and *Rspo3* KO (*n* = 6 mice) mice treated with 1.5% DSS for 5 days. Data represent mean ± SD; Student’s *t*-test (two-tailed) was applied in all cases. *p < 0.05, **p < 0.01, ***p < 0.001, ****p < 0.0001. Scale bar = 100 µm unless indicated otherwise. Source data are provided as a source data file

Our data indicate that the Wnt^+^ cell compartment expands during crypt regeneration in the colon and since Rspo3 restricts this compartment during homeostasis, we asked how expression of *Rspo3* is affected by DSS. Using a recently published RNAseq data set from stromal myofibroblasts of untreated and DSS-treated mice^17^ and applying pseudo-bulk analysis, we noticed a significant increase of *Rspo3* expression in the stroma (Fig. 7d). Using ISH and image analysis we confirmed that after DSS was discontinued, overall expression of *Rspo3* increased (Fig. 7a–c).

To address the role of Rspo3 during the regenerative state, we induced mild colitis by using 1.5% of DSS (Fig. 7e). At the time when DSS was discontinued, we did not observe a significant difference in weight loss between WT and *Rspo3* KO animals. However, while the WT animals recovered from injury, *Rspo3* KO mice progressively lost weight and were unable to re-establish a normal crypt architecture (Fig. 7f, g). This contrasts starkly with our observation that during homeostatic conditions an almost normal crypt architecture is maintained even in *Rspo* KO animals in which Lgr5 cells have been depleted. Thus, the elevated levels of Rspo we observed during regeneration appear necessary to enhance Wnt signaling, most likely via Lgr4 receptors, in differentiated cells.

## Discussion

It has been demonstrated previously that loss of Lgr5^+^ stem cells in the gastrointestinal tract leads to activation of different cell populations that can regenerate the crypts^3,18^. Most of this work has been done in the small intestine, where highly proliferative progenitors^4^, as well as differentiated cell populations including Bmi1^+^^18^ and Paneth cells^19^ can act as stem cells after Lgr5^+^ cell loss. In the colon, secretory progenitors marked by Atoh1 have been shown to give rise to full crypts in the context of injury^5^. Instead of focusing on a specific cell type, here we focused on Axin2 as a marker of Wnt signaling and characterized the dynamic alteration of its expression after injury. Our data indicate that re-activation of Wnt signaling in differentiated cells is driven by Rspo3, and made possible because the majority of differentiated cells express the Rspo3 receptor Lgr4. This also explains the high plasticity seen in gastrointestinal crypts.

We find that Lgr5^+^ cells are lost during colitis, confirming previous reports in different models^20^. Consistent with this, it was recently demonstrated that Lgr5^+^ cells are dispensable for crypt recovery from DSS colitis^6^. Interestingly, not Lgr5 cells per se are responsible for epithelial regeneration but rather Rspo3, the main driver of the Lgr5^+^ cell signature, which controls the size of the Lgr5^+^ cell pool by inducing their self-renewal^10^. This observation not only demonstrates an additional essential function for Rspo3, which is to reprogram differentiated cells during colonic wound healing, but also provides evidence that ultimately the cellular environment, rather than its phenotypic identity, determines the fate of the cell. Although it is well established that Lgr5 marks stem cells, it remains unclear whether Axin2^+^/Lgr5^+^ colon epithelial cells are truly a different cell type with tissue stem cell properties or whether increased Wnt/Rspo signaling promotes Lgr5 expression and confers increased proliferative capacity. Our data indicate that various different Lgr5^−^ cells can respond to Rspo3 and that high Wnt/Rspo induces expression of Wnt target genes and proliferation—demonstrating that these functions can be induced without Lgr5. While we clearly demonstrate that Lgr5 expression is Rspo3-dependent, we also find that many but not all genes characteristic for Lgr5^+^ cells are driven by Rspo3. Therefore, it is likely that Rspo acts in concert with other signals to control the state and function of Lgr5^+^ cells.

Lgr5^+^ cells show full recovery upon depletion^3^, suggesting that this is due to signals from the stem cell niche that remain unaltered and simply reprogram neighboring cells to stem cells. In this context Wnt signaling has been postulated as a critical signal and recent reports have determined the cellular identity of stromal cells that express Wnt ligands^7,21^. Rspo3 is also expressed in several different types of stromal cells, including Wnt-producing telocytes^22^. Our data using Lgr5DTR mice show that Rspo3 from the stroma is required for regeneration of Lgr5^+^ cells upon depletion. However, in the context of a more severe injury, where the cells with active Wnt signaling are lost, the niche does not remain static but undergoes marked remodeling^17,23^, which is accompanied by an increased expression of Rspo3. This demonstrates that stromal cells can rapidly adapt to injury, which is critical for an efficient recovery of wounded epithelium, as it leads to re-induction of Wnt signaling in differentiated cells.

While Rspo3 is crucial for epithelial regeneration, it is likely that other signals act in concert with Wnt and Rspo3 during colonic epithelial repair. Inflammatory cells, including macrophages and T cells, along with altered morphogen signals can drive epithelial regeneration and represent an emerging field of investigation^24^. In addition to cellular factors, changes in the composition of the extracellular matrix may also have a critical impact on epithelial recovery. Likewise, YAP activation driven by collagen remodeling induces alternative signaling reminiscent of fetal development in the context of DSS colitis^25^. R-spondins are characterized by the presence of a thrombospondin domain, which can bind matrix glycosaminoglycans and proteoglycans. Thus, it will be important to study the interplay between extracellular matrix and Rspo molecules in the healthy state and in the context of injury.

A recent landmark study provided the proof of concept that differentiated surface cells can de-differentiate during Wnt-driven carcinogenesis. Mutations of β-catenin that lead to deregulated enhanced activation of Wnt signaling in differentiated cells, in cooperation with the NF-κB pathway, can induce tumor development^26^. We find here that a similar phenomenon occurs as part of an endogenous program required for crypt regeneration upon injury. While this signal is crucial for regeneration, its limitation is important to prevent uncontrolled growth and carcinogenesis. Experimental^27^ and clinical^28^ data suggest that uncontrolled expression of Rspo3 drives malignant transformation, although it is not yet clear to what extent recruitment of differentiated cells plays a role in this.

Aberrant expression of the stem cell marker Olfm4 in the surface of crypts was shown to occur in human IBD^26^, indicating that activation of regenerative pathways similar to what we observe here may occur in patients with chronic colonic injury. Recruitment of differentiated cells back into the stem cell compartment bears an inherent risk of epithelial transformation. Luminal bacteria, bacterial toxins, and metabolites have the capacity to induce DNA damage that can lead to loss of genetic integrity^29^ and have been linked to colorectal cancer^30^. Crypt stem cells are not only located in a compartment that is removed from the influence of luminal contents, but other mechanisms contributing to their protection have also recently been revealed: Accordingly, secretory cells located in direct proximity, such as Paneth cells^31^ in the small intestine or c-kit^+^ cells in the colon^32^, produce a variety of antimicrobial factors that shield stem cells from invading bacteria. In addition, colon stem cells were recently shown to be protected from bacterial metabolites^16^. By contrast, differentiated cells at the crypt surface are not protected by such mechanisms, and are normally shed into the lumen within hours or days. Recruitment of such potentially damaged cells to act as stem cells in the context of injury, as shown here in the case of severe injury, may account for the increased risk of colorectal carcinogenesis observed during chronic colonic epithelial damage, such as ulcerative colitis^33^.

## Methods

### Mouse experiments

All procedures involving animals were approved by the institutional and legal authorities at the Max Planck Institute for Infection Biology (LaGeSo Berlin). *Axin2CreErt2/Rosa26-tdTomato* mice were used for lineage tracing of cells that derived from Axin2-expressing cells. *Lgr5DTR* mice^3^ used in this study were provided by Genetech. *Rspo3*^*fl/fl*^ mice^34^ were a kind gift from John Cobb. *Myh11CreErt2*^35^ mice were a kind gift from Stefan Offermanns. To generate *Krt20CreErt2/Rosa26-tdTomato* mice, we bred *Krt20CreErt2* mice (Jackson Laboratories) to *Rosa26-tdTomato* mice to follow the fate of KRT20^+^ cells upon tamoxifen injection.

*Lgr5CreErt2/Rosa26-tdTomato* mice were generated by breeding of *Lgr5–eGFP–IRES-CreErt2 (Lgr5eGFP)*^2^ to *Rosa26-tdTomato* mice.

To generate conditional KO mice with depletion of Rspo3 in myofibroblasts, we bred *Rspo3*^*fl/fl*^ mice to *Myh11CreErt2* mice, while *Myh11CreErt2/Rspo3*^*+/+*^ littermates served as controls. To additionally deplete Lgr5^+^ cells, the mice were bred to *Lgr5DTR* mice to generate *Lgr5DTR/Myh11CreErt2/Rspo3*^*fl/fl*^ animals. All animals were maintained in autoclaved micro-isolator cages and provided with sterile drinking water and chow ad libitum. Male, 6–8-week-old mice were used for this study.

### Lineage tracing and Rspo3 depletion

Tamoxifen (Sigma) was injected intraperitoneally into mice either as a single dose or on 3 consecutive days (4 mg/25 g body weight, diluted in 200 μl corn oil) at the indicated time points to deplete Rspo3 expression or induce lineage tracing.

### Lgr5 cell depletion

DT (Sigma, 50 μg/kg body weight) was injected intraperitoneally at indicated time points into mice carrying the *Lgr5DTReGFP* allele. Mice were euthanized 24 h or 1 week after injection of a single dose or three doses of DT. To analyze the role of Axin2 in the absence of Lgr5^+^ cells, *Lgr5DTR/Axin2CreErt2/Rosa26-tdTomato* mice were treated with tamoxifen and DT on the same day. In addition, follow-up doses of DT were administered at days 2 and 3 after tamoxifen. The same treatment scheme was applied for *Lgr5DTR/Myh11Rspo3*^*fl/fl*^ mice to investigate the role of Rspo3 for Lgr5^+^ cell recovery.

### DSS treatment

Dextran sodium sulfate (MP Biomedical, 36,000–50,000 MW, CAS number 9011-18-1) was dissolved in autoclaved tap water to obtain concentrations of 1.5–2.5%. The solution was then sterile-filtered and provided to mice for the indicated period from 3 to 7 days. For recovery experiments DSS was replaced by normal drinking water. At the end of the experiment mice were sacrificed, colon was isolated, and the length of the colon was measured. Samples for immunofluorescence, single-molecule ISH, and RNA isolation were collected and processed.

### Tissue processing

For preserving and visualizing of fluorescent proteins expressed in mice, such as eGFP in *Lgr5eGFP* mice or TdTomato in *Rosa26-TdTomato* animals, colon tissue was opened longitudinally and fixed for 1 h with 4% PFA. Tissue was then washed with PBS, embedded in 4% agar and sectioned using a vibratome (Leica). Vibratome sections (100–300 µm thick) were permeabilized in PBS with 3% BSA, 1% saponin, and 1% Triton X-100 prior to staining. The samples were stained overnight with DAPI for nucleus visualization and with phalloidin to visualize cell boundaries. For paraffin embedding colon pieces were flushed with PBS and fixed in 2% PFA for 1 day—paraffin embedding and sectioning, as well as H&E staining, which was performed by the Charité Core Unit Immunopathology for Experimental Models.

### Immunofluorescence

Paraffin-embedded sections were rehydrated and following an antigen retrieval step incubated with primary antibodies against Ki67, E-cadherin, and Aquaporin 8 (detailed information indicated in Supplementary Table 1) overnight, followed by a washing step and incubation with secondary antibody (Supplementary Table 2) for 1 h. Samples were imaged with a Leica Sp8 confocal microscope.

### TUNEL assay

Paraffin-embedded sections were analyzed using the Click-iT™ Plus TUNEL Assay for In Situ Apoptosis Detection, according to the manufacturer’s instructions (ThermoFisher Scientific). DNase I-treated samples were used as positive controls.

### Single-molecule RNA ISH

Tissue sections cut at 5-μm thickness were processed for RNA in situ detection using an RNAscope Red Detection Kit according to the manufacturer’s instructions (Advanced Cell Diagnostics, Hayward, CA, USA). Positive and negative control probes were used for each experiment according to the manufacturer’s instructions. Probes used in this study are indicated in Supplementary Table 3.

### Quantitative RT-PCR

RNA was extracted from snap-frozen colon tissue using the RNAeasy RNA Purification Kit (Qiagen) including on-column DNase digestion. qPCR was performed using a Power SYBR Green RNA-to-CT 1-Step Kit (Applied Biosystems) according to the manufacturer’s instructions. Reactions were performed in 25 μl containing 50 ng RNA, 12.5 μl SYBR Green mix, 0.16 μl RT mix, and 0.2 μM primer (for sequences see Supplementary Table 4). Program: 30 min at 48 °C; 10 min at 95 °C; followed by 40 cycles of 15 s at 95 °C/60 s at 60 °C. For each oligonucleotide pair and RNA sample, the reaction was performed in triplicate. The amplification plots obtained from the RT-PCR were analyzed with StepOne™ Real-Time PCR Software v2.2. The expression levels of the target genes were normalized to the levels of glyceraldehyde-3-phosphate dehydrogenase gene expression in each individual sample.

### Cell sorting

For the comparison of Axin2^+^ and Axin2^–^ cells, lineage tracing was induced for 24 h in three *Axin2CreErt2/Rosa26-tdTomato* mice as described above. The mice were sacrificed and the colon was isolated. Colonic tissue was washed, cut into small pieces and incubated in buffer containing 10 mM EDTA and 0.5 mM DTT for 30 min. After incubation, samples were shaken vigorously. Tissue fragments were allowed to settle by gravity and the supernatant, including the crypts, was washed and resuspended in cell dissociation solution containing TrypleExpress (Gibco) and Accumax (Innovative Cell Technologies). After incubation at 37 °C for 5 min, cells were washed in advanced DMEM medium containing 10% FCS and labeled with a conjugated antibody against epithelial surface antigen (ESA) in PBS with 0.5% BSA and 2 mM EDTA. After an additional washing step, cells were resuspended in PBS supplemented with HEPES/FCS and filtered through a 40 µm mesh. Cells were analyzed for tdTomato and ESA expression by flow cytometry using the BD FACSAria II flow cytometer. DAPI was used to distinguish between live and dead cells. Cells were directly sorted into TRIzol solution.

### Single-cell RNAseq data analysis

Stromal scRNAseq data from colon of untreated and DSS-treated mice was used from the data set generated by the Simmons group^17^. Fibroblast single cell populations were isolated in silico, discarding other cell types (glial, endothelial, and pericyte populations) and a pseudo-bulk for each sample was generated by aggregating raw read counts from the selected cellular barcodes. DESeq2 R package^36^ was used to compute library size factors, normalise the data and perform differential expression analysis using negative binomial Wald’s test. Benjamini–Hochberg correction was used to estimate false discovery rate (FDR). The code and data to recreate the analysis are deposited under https://github.com/agneantanaviciute/Rpos3pseudobulk.

Characterization of colonic epithelial cells using RNAseq was done using the Tabula Muris data set^13^. Data was extracted and re-analyzed for expression of specific markers. Clusters that express *Lgr5* were identified and named as Lgr5^+^/Axin2^+^ cells, whereas the remaining cluster of Lgr5^−^ cells that expressed Axin2 was named Lgr5^−^/Axin2^+^. Expression of genes in the defined clusters was visualized using violin plots.

### Microarray analysis

For analysis of *Rspo3* KO and corresponding *Rspo3*^+/+^ littermates, RNA from the colon was isolated using the RNeasy Mini Kit (Qiagen) as described above. For the comparison of Axin2^+^ and Axin2^−^ cells, RNA was isolated by the Trizol (Invitrogen) method following the manufacturer’s protocol after direct sorting into Trizol. Microarray experiments were performed as independent dual-color dye-reversal color-swap hybridizations using two biological replicates per group for the *Rspo3* KO mice. For the microarray of Axin2^+^ and Axin2^−^ cells the subpopulations were sorted and cells from three mice were pooled for microarray analysis.

Quality control and quantification of total RNA was carried out using an Agilent 2100 Bioanalyzer (Agilent Technologies) and a NanoDrop 1000 UV–Vis spectrophotometer (Kisker). RNA labeling was performed with a dual-color Quick-Amp Labeling Kit (Agilent Technologies). In brief, mRNA was reverse transcribed and amplified using an oligo-dT-T7 promoter primer, and the resulting cRNA was labeled with cyanine 3-CTP or cyanine 5-CTP. After precipitation, purification, and quantification, 1.25 μg of each labeled cRNA was fragmented and hybridized to whole-genome mouse 4 × 44 K multipack microarrays (Agilent-014868, whole mouse genome 4 × 44 K microarray kit) according to the manufacturer’s protocol (Agilent Technologies). Scanning of microarrays was performed at 5 μm resolution using a G2565CA high-resolution laser microarray scanner (Agilent Technologies) with extended dynamic range (XDR). Microarray image data were analyzed with image analysis/feature extraction software G2567AA version A.11.5.1.1 (Agilent Technologies) using default settings and the GE2_1105_Oct12 extraction protocol. The extracted MAGE-ML files were analyzed further with Rosetta Resolver Biosoftware, build 7.2.2 SP1.31 (Rosetta Biosoftware). Ratio profiles comprising single hybridizations were combined in an error-weighted fashion to create ratio experiments. A 1.5-fold change expression cut-off for ratio experiments was applied together with anti-correlation of dye-swapped ratio profiles, rendering the microarray analysis highly significant (*p* < 0.01), robust, and reproducible. Microarray data have been deposited in the gene expression omnibus (GEO; [https://www.ncbi.nlm.nih.gov/geo/]) of the National Center for Biotechnology Information under accession number GSE115752.

### GSEA analysis

We performed GSEA on genes pre-ranked by gene expression-based *t*-score between colon epithelium isolated from *Rspo* KO and *Rspo* WT controls and between Axin^+^ and Axin^−^ cells using the fgsea R package^37^ with 5000 permutations. We used gene sets from MSigDB v6.2^38,39^ and a gene set of a stem cell signature obtained from Lgr5^+^ cells in intestinal crypts published previously^14^. *P*-values were adjusted for multiple testing by a global FDR according to the method described by Benjamini and Hochberg. R version 3.4 was used and can be obtained from https://cran.r-project.org/, while the fgsea package can be retrieved from https://www.bioconductor.org.

The computational code for GSEA analysis of microarray in this manuscript data can be accessed under https://github.com/MPIIB-Department-TFMeyer/Harnack_et_al_Rspo3_colon.

### Statistics

No statistical methods were used to predetermine sample size. Mouse experiments were performed on at least *n* = 3 biological replicates, except for the microarray analysis, in which two biological replicates were used. No mice were excluded from experiments. All data are displayed as mean ± SD for the various groups. Statistics are based on ‘*n*’ biological replicates. For the comparisons of two groups, a *t*-test or non-parametric test was performed. Non-parametric testing was performed if a normal distribution could not be assumed. All analyses of statistical significance were calculated and displayed compared with the reference control group unless otherwise stated. For data visualization and statistical analysis, Graph Pad Prism 7 software was used.

### Reporting summary

Further information on research design is available in the Nature Research Reporting Summary linked to this article.

## Supplementary information

Supplementary Information

Reporting Summary

Source Data

## Data Availability

The microarray data from this manuscript have been deposited in the National Centre for Biotechnology Information Omnibus (GEO) under accession code GSE115752. Quantitative data supporting the findings of this study are available within the paper and its supplementary information files. All other data supporting these findings are available from corresponding author upon reasonable request. The source data underlying Figs 1b, c, h, i 2a, b, c, f, h, 3a, b, f, h, j, 4b, d, f, h, 5b, g, 6c, d, e, g, 7c, e, g and Supplementary Figs. S2b, S3a, S5a, b are provided in the Source Data File.

## Code availability

The computational code for GSEA analysis of microarray in this manuscript plus all additional content required can be accessed under https://github.com/MPIIB-Department-TFMeyer/Harnack_et_al_Rspo3_colon. The code and data to recreate the pseudobulk analysis of single cell RNAseq data from stromal colonic cells are deposited under https://github.com/agneantanaviciute/Rpos3pseudobulk.

## References

[1] Barker N., van Es J.H., Jaks V., Kasper M., Snippert H., Toftgard R., Clevers H. (2008). Very Long-term Self-renewal of Small Intestine, Colon, and Hair Follicles from Cycling Lgr5+ve Stem Cells. Cold Spring Harbor Symposia on Quantitative Biology.

[2] Barker N (2007). Identification of stem cells in small intestine and colon by marker gene Lgr5. Nature.

[3] Tian Hua, Biehs Brian, Warming Søren, Leong Kevin G., Rangell Linda, Klein Ophir D., de Sauvage Frederic J. (2011). A reserve stem cell population in small intestine renders Lgr5-positive cells dispensable. Nature.

[4] Tetteh PW (2016). Replacement of Lost Lgr5-positive stem cells through plasticity of their enterocyte-lineage daughters. Cell Stem Cell.

[5] Tomic G (2018). Phospho-regulation of ATOH1. Cell Stem Cell.

[6] Castillo-Azofeifa, D. et al. Atoh1(+) secretory progenitors possess renewal capacity independent of Lgr5(+) cells during colonic regeneration. *EMBO J*. 10.15252/embj.201899984 (2019). 10.15252/embj.201899984 PMC6376326 30635334

[7] Degirmenci Bahar, Valenta Tomas, Dimitrieva Slavica, Hausmann George, Basler Konrad (2018). GLI1-expressing mesenchymal cells form the essential Wnt-secreting niche for colon stem cells. Nature.

[8] Kabiri Z (2014). Stroma provides an intestinal stem cell niche in the absence of epithelial Wnts. Development.

[9] de Lau W (2011). Lgr5 homologues associate with Wnt receptors and mediate R-spondin signalling. Nature.

[10] Yan KS (2017). Non-equivalence of Wnt and R-spondin ligands during Lgr5+ intestinal stem-cell self-renewal. Nature.

[11] Sigal M (2017). Stromal R-spondin orchestrates gastric epithelial stem cells and gland homeostasis. Nature.

[12] Greicius G (2018). PDGFRalpha(+) pericryptal stromal cells are the critical source of Wnts and RSPO3 for murine intestinal stem cells in vivo. Proc. Natl Acad. Sci. USA.

[13] Tabula Muris C (2018). Single-cell transcriptomics of 20 mouse organs creates a Tabula Muris. Nature.

[14] Munoz J (2012). The Lgr5 intestinal stem cell signature: robust expression of proposed quiescent ‘+4’ cell markers. EMBO J..

[15] Chassaing B, Aitken JD, Malleshappa M, Vijay-Kumar M (2014). Dextran sulfate sodium (DSS)-induced colitis in mice. Curr. Protoc. Immunol..

[16] Kaiko GE (2016). The colonic crypt protects stem cells from microbiota-derived metabolites. Cell.

[17] Kinchen J (2018). Structural remodeling of the human colonic mesenchyme in inflammatory bowel disease. Cell.

[18] Yan KS (2012). The intestinal stem cell markers Bmi1 and Lgr5 identify two functionally distinct populations. Proc. Natl Acad. Sci. USA.

[19] Yu S (2018). Paneth cell multipotency induced by notch activation following injury. Cell Stem Cell.

[20] Santos AJM, Lo YH, Mah AT, Kuo CJ (2018). The intestinal stem cell niche: homeostasis and adaptations. Trends Cell Biol..

[21] Shoshkes-Carmel M (2018). Subepithelial telocytes are an important source of Wnts that supports intestinal crypts. Nature.

[22] Aoki R (2016). Foxl1-expressing mesenchymal cells constitute the intestinal stem cell niche. Cell. Mol. Gastroenterol. Hepatol..

[23] Stzepourginski I (2017). CD34+ mesenchymal cells are a major component of the intestinal stem cells niche at homeostasis and after injury. Proc. Natl Acad. Sci. USA.

[24] Naik S, Larsen SB, Cowley CJ, Fuchs E (2018). Two to Tango: dialog between immunity and stem cells in health and disease. Cell.

[25] Yui S (2018). YAP/TAZ-dependent reprogramming of colonic epithelium links ECM remodeling to tissue regeneration. Cell Stem Cell.

[26] Schwitalla S (2013). Intestinal tumorigenesis initiated by dedifferentiation and acquisition of stem-cell-like properties. Cell.

[27] Hilkens J (2017). RSPO3 expands intestinal stem cell and niche compartments and drives tumorigenesis. Gut.

[28] Seshagiri S (2012). Recurrent R-spondin fusions in colon cancer. Nature.

[29] Chumduri C, Gurumurthy RK, Zietlow R, Meyer TF (2016). Subversion of host genome integrity by bacterial pathogens. Nat. Rev. Mol. Cell Biol..

[30] Arthur JC (2012). Intestinal inflammation targets cancer-inducing activity of the microbiota. Science.

[31] Clevers HC, Bevins CL (2013). Paneth cells: maestros of the small intestinal crypts. Annu. Rev. Physiol..

[32] Rothenberg ME (2012). Identification of a cKit(+) colonic crypt base secretory cell that supports Lgr5(+) stem cells in mice. Gastroenterology.

[33] Swidsinski A (2002). Mucosal flora in inflammatory bowel disease. Gastroenterology.

[34] Neufeld S (2012). A conditional allele of Rspo3 reveals redundant function of R-spondins during mouse limb development. Genesis.

[35] Herring BP, Hoggatt AM, Burlak C, Offermanns S (2014). Previously differentiated medial vascular smooth muscle cells contribute to neointima formation following vascular injury. Vasc. Cell.

[36] Love MI, Huber W, Anders S (2014). Moderated estimation of fold change and dispersion for RNA-seq data with DESeq2. Genome Biol..

[37] Sergushichev, A. An algorithm for fast preranked gene set enrichment analysis using cumulative statistic calculation. Preprint at 10.1101/060012 (2016).

[38] Subramanian A (2005). Gene set enrichment analysis: a knowledge-based approach for interpreting genome-wide expression profiles. Proc. Natl Acad. Sci. USA.

[39] Liberzon A (2011). Molecular signatures database (MSigDB) 3.0. Bioinformatics.

