# Supplementary material for: R-spondin 3 promotes stem cell recovery and epithelial regeneration in the colon
Source: Nat Commun. 2019 Sep 25;10:4368. doi: 10.1038/s41467-019-12349-5 (PMC6761174; doi:10.1038/s41467-019-12349-5)
Supplement: Supplementary file 1 — Supplementary Information [file 41467_2019_12349_MOESM1_ESM.pdf]

R-spondin 3 promotes stem cell recovery and epithelial regeneration in the colon

Harnack et al.

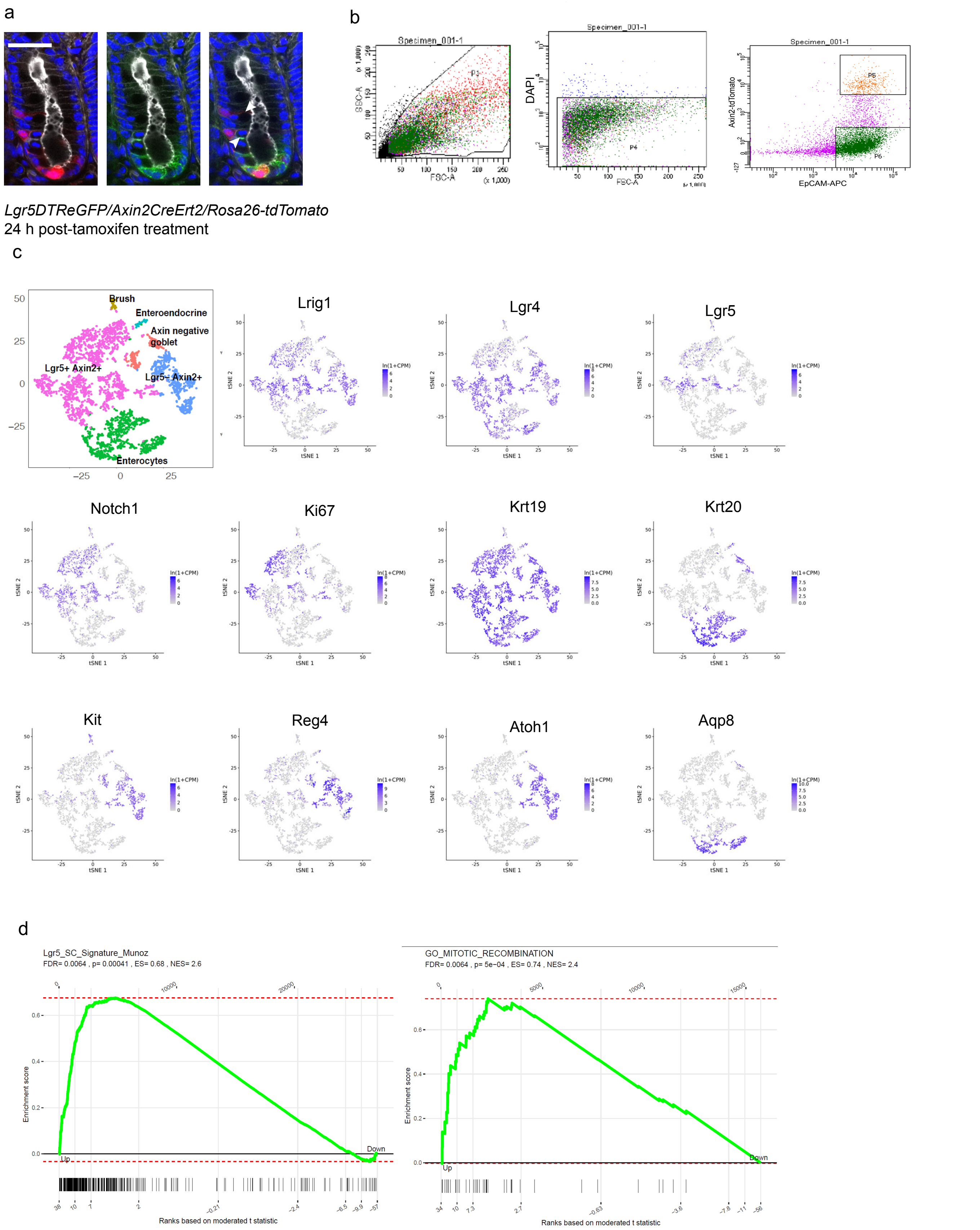

Supplementary Figure 1. Axin2+ cells show a stem cell related expression pattern

a, Co-localization of Axin2 and Lgr5 in *Lgr5DTReGFP/Axin2CreErt2/Rosa26tdTomato* mice 24 h after tamoxifen injection. Double positive cells are indicated by arrows, Axin2^{+}/Lgr5^{-} cells are indicated by arrow heads (scale bar=25µm).

b, FACS scheme for sorting of Axin2^{+} vs Axin2^{-} cells 24 h post-tamoxifen injection in *Axin2CreErt2/Rosa26-tdTomato* mice. Axin2^{+} cells are labeled by tdTomato. EpCAM-APC was used to label epithelial cells. Live-dead was distinguished using DAPI.

c, t-SNE plots of single cell RNAseq data.

d, GSEA analysis of genes differentially expressed in Axin2^{+} vs Axin2^{-} cells in comparison to the previously published small intestinal Lgr5^{+} stem cell signature gene set (cells pooled from 3 mice were used) and a mitotic recombination-related signature gene set obtained from MSigDB (cells pooled from 3 mice were used).

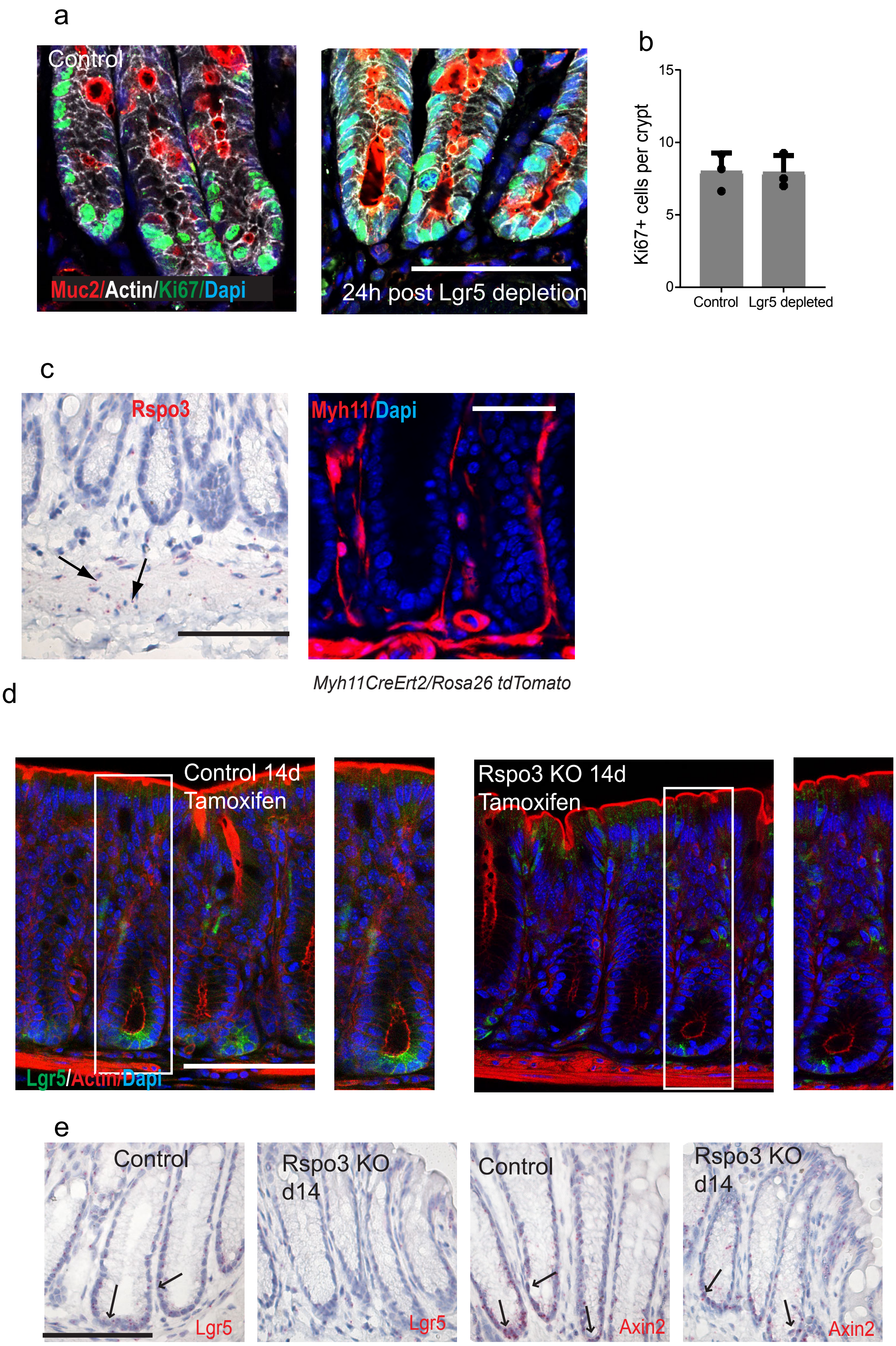

Supplementary Figure 2.

a, Confocal images of colon tissue from control mice and *Lgr5DTR* mice treated 3 times with DT after 24 h recovery stained for Muc2 (red) and Ki67 (green).

b, Quantification of Ki67 cells per crypt (control n=3 mice, *Lgr5DTR* n=3 mice) All bar charts present data as mean ±SD.

c, Single-molecule ISH for Rspo3 in the colon alongside confocal microscopy image of colon tissue from a Myh11CreErt2/Rosa26-tdTomato mouse (scale bar=50µm).

d, Confocal microscopy images from the colon of an *Lgr5DTReGFP/Myh11CreErt2/Rspo3*$^{fl/fl}$ and an *Lgr5DTReGFP/Myh11CreErt2/Rspo3*$^{+/+}$ mouse treated with a single dose of tamoxifen and DT 7d before sacrifice.

e, Single-molecule in situ hybridization for Lgr5 and *Axin2* in *Rspo* KO and WT mice. KO was induced 14 d prior to sacrifice. Scale bar=100µm if not indicated otherwise. All experiments were performed in at least 3 biological replicates Source data are provided as a source data file.

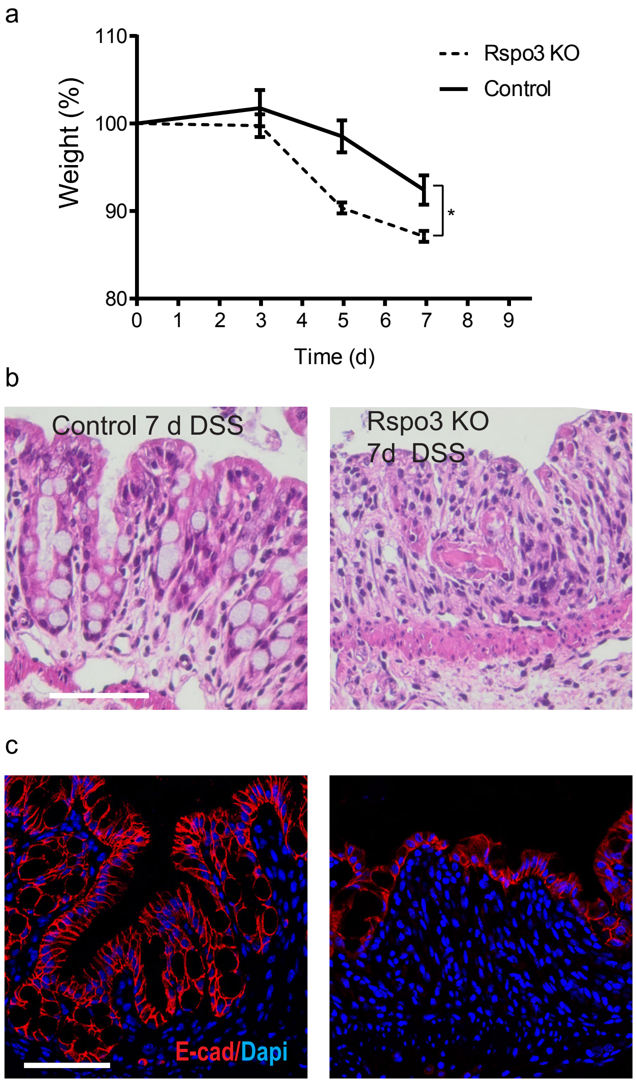

Supplementary Figure 3.

a, Weight curves of control (n=5 mice) and Rspo3 KO (n=6 mice) mice during DSS treatment; *: $p<0.05$, Student´s t-test (two-tailed).

b, H&E staining of colon tissue from a DSS-treated control mouse and a DSS-treated *Rspo3* KO mouse that shows an almost complete loss of the epithelial lining. Mice were sacrificed at 7 d of DSS treatment.

c, Confocal microscopy of the colon epithelium detected by immunofluorescence labelling of E-cadherin in a DSS-treated control mouse and a DSS-treated *Rspo3* KO mouse sacrificed at 7 d of DSS treatment. All experiments were performed in at least 3 biological replicates. Data represent mean ± SD analyzed by Student's t-test (two-tailed). Scale bar = 100 µm. Source data are provided as a source data file.

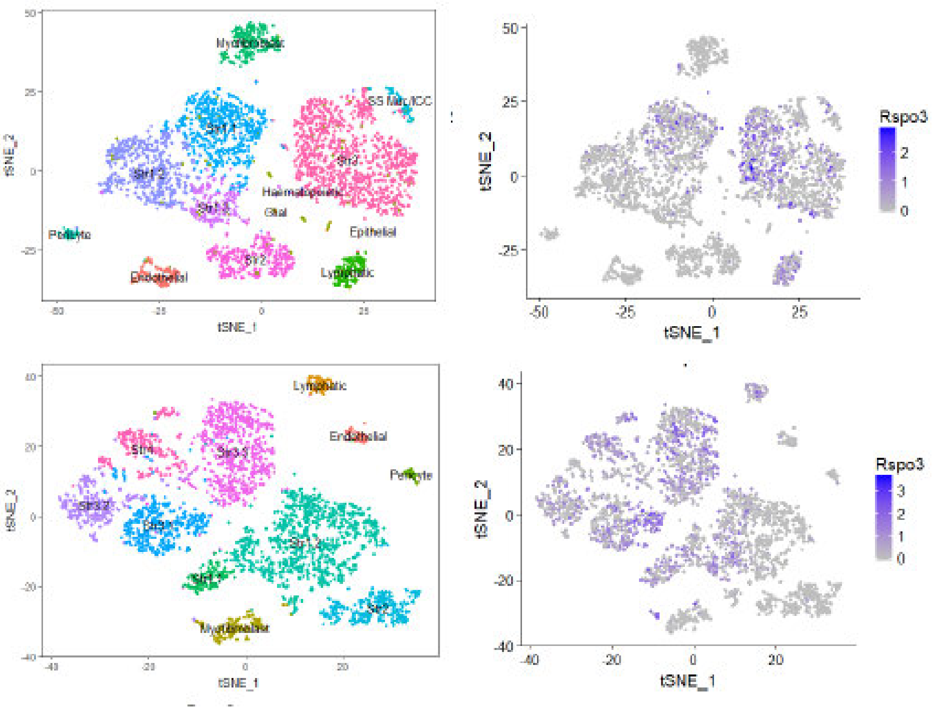

Supplementary Figure 4.
t-SNE Plots of RNAseq Data for Rspo3 from untreated control animals (top row) and DSS-treated animals (bottom row).

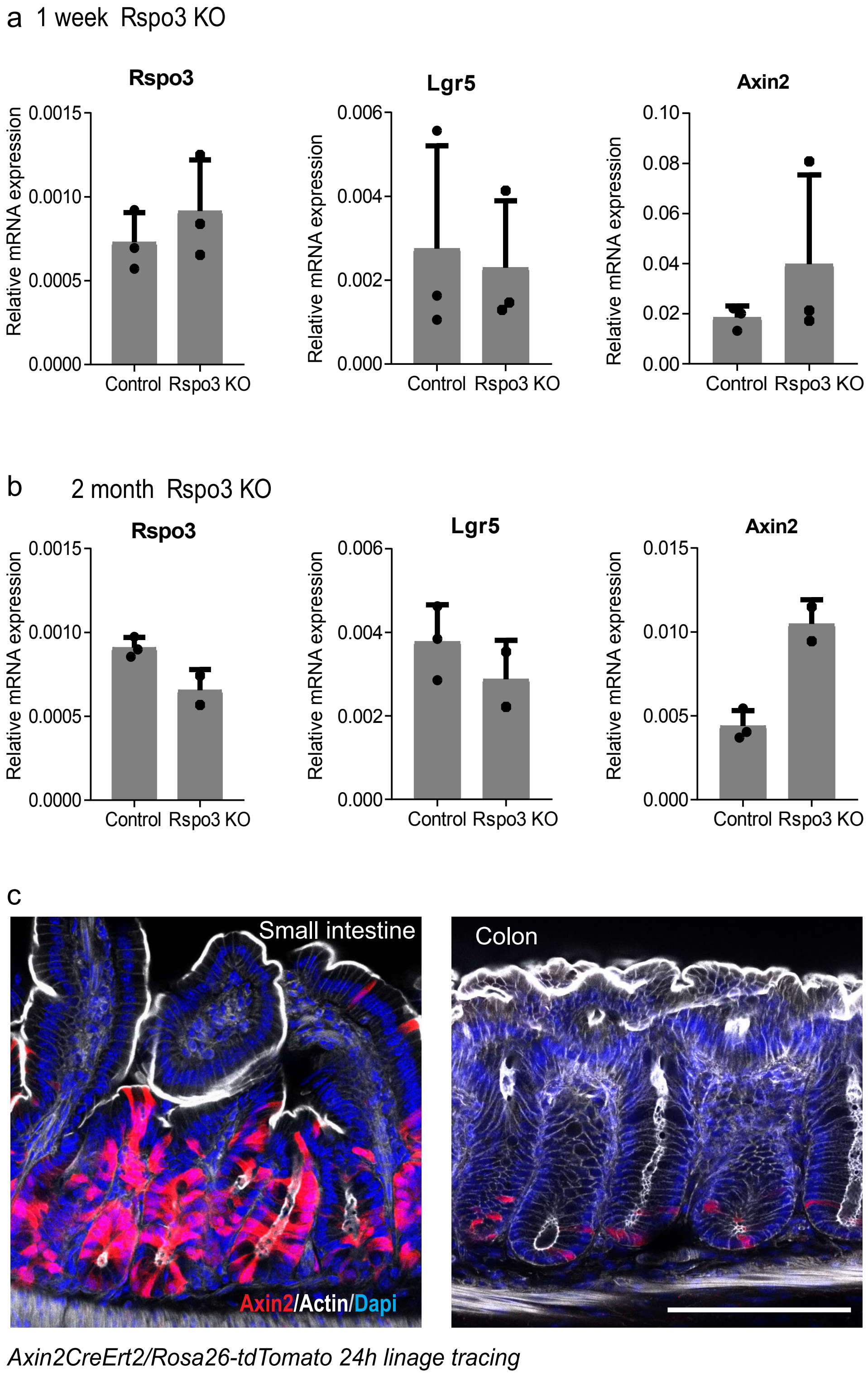

Supplementary Figure 5.

a, qPCR results for *Rspo3*, *Lgr5* and *Axin2* in small intestine after 1 week of *Rspo3* KO (control n=2, KO n=3 mice).

b, qPCR results for *Rspo3*, *Lgr5* and *Axin2* in small intestine after 2 months of *Rspo3* KO(control n=2, KO n=3 mice).

c, Confocal microscopy images from small intestine and colon of an *Axin2CreErt2/Rosa26-tdTomato* mouse 24 h after tamoxifen injection. Scale bar=100µm. All bar charts present data as mean ±SD. Source data are provided as a source data file.

Supplementary Table 1: Primary antibodies used

Antibody	Host	Species	Company	Cat. number	Concentration
E-cadherin	Mouse	Human	BD Pharmingen	610181	1:300
Aquaporin 8	Rabbit	Polyclonal	Bioss	Bs-6786R	1:100
UEAI			Vector Laboratories	FL-1061	1:100
Ki-67	Rat	Mouse	ThermoFisher	11-5698-82	1:100
Ki-67	Rabbit	Mouse	Cell Signaling	9192S	1:100
Krt20	Rabbit	Mouse	Cell Signaling	13063	1:200
DAPI			Roche	10236276001	1:300
EpCAM		Mouse	Miltenyi Biotech	130-102-234	1:10

Supplementary Table 2: Secondary antibodies used

Host	Species	Label	Company	Cat. number	Concentration
Donkey	Mouse	Alexa Flour647	Dianova	715-605-150	1:250
Donkey	Rabbit	Alexa Flour488	Dianova	711-546-152	1:250
Donkey	Rabbit	Cy3	Dianova	711-165-152	1:250
Phalloidin		AlexaFlour647	Invitrogen	A22287	1:100

Supplementary Table 3: RNAscope probes

Gene	Order no.	Lot	Target Region
Axin2	400331	16347A	330-1287
Lgr4	318321	18053A	1973-2895
Lgr5	312171	16351A	2165-3082
Rspo3	402011	16321A	731-2164
Krt20	402301	16344B	307-1287
pos.control (ppib)	313911	16298A	98 - 856
neg.control (DapB)	310043	16302A	414-862

Supplementary Table 4: Primer sequences

Primer	Direction	Species	Sequence	Company
mGAPDH	Forward	Mouse	TCACCATCTTCCAGGAGCG	Sigma
mGAPDH	Reverse	Mouse	AAGCAGTTGGTGGTGCAGG	Sigma
mLGR5	Forward	Mouse	CCTACTCGAAGACTTACCCAGT	Sigma
mLGR5	Reverse	Mouse	GCATTGGGGTGAATGATAGCA	Sigma
mAxin2	Forward	Mouse	TGACTCTCCTTCCAGATCCCA	Sigma
mAxin2	Reverse	Mouse	TGCCCACACTAGGCTGACA	Sigma
mRspo1	Forward	Mouse	ACAGAGGCGGATCAGTGC	Sigma
mRspo1	Reverse	Mouse	GGCAACCGTTGACTTCTGAAC	Sigma
mRspo2	Forward	Mouse	CAGCCCGAGAGACGCGAG	Sigma
mRspo2	Reverse	Mouse	ACGGTGAACTGGCACGATCC	Sigma
mRspo3	Forward	Mouse	TTGACAGTTGCCCAGAAGGG	Sigma
mRspo3	Reverse	Mouse	CTGGCCTCACAGTGTACAATACT	Sigma
mRspo4	Forward	Mouse	TGTACCGAAGGAAGAAGCAAGC	Sigma
mRspo4	Reverse	Mouse	CCCACATTTCTTGCACCTGTTG	Sigma
