# Supplementary material for: R-spondin 3 promotes stem cell recovery and epithelial regeneration in the colon
Source: Nat Commun. 2019 Sep 25;10:4368. doi: 10.1038/s41467-019-12349-5 (PMC6761174; doi:10.1038/s41467-019-12349-5)
Supplement: Supplementary file 2 — Reporting Summary [file 41467_2019_12349_MOESM2_ESM.pdf]

nature research

Corresponding author(s): Michael Sigal, Thomas F Meyer

Last updated by author(s): 24.07.2019

# Reporting Summary

Nature Research wishes to improve the reproducibility of the work that we publish. This form provides structure for consistency and transparency in reporting. For further information on Nature Research policies, see Authors & Referees and the Editorial Policy Checklist.

## Statistics

For all statistical analyses, confirm that the following items are present in the figure legend, table legend, main text, or Methods section.

| n/a | Confirmed | |
|---|---|---|
| ☐ | ☒ | The exact sample size ($n$) for each experimental group/condition, given as a discrete number and unit of measurement |
| ☐ | ☒ | A statement on whether measurements were taken from distinct samples or whether the same sample was measured repeatedly |
| ☐ | ☒ | The statistical test(s) used AND whether they are one- or two-sided<br>*Only common tests should be described solely by name; describe more complex techniques in the Methods section.* |
| ☐ | ☒ | A description of all covariates tested |
| ☐ | ☒ | A description of any assumptions or corrections, such as tests of normality and adjustment for multiple comparisons |
| ☐ | ☒ | A full description of the statistical parameters including central tendency (e.g. means) or other basic estimates (e.g. regression coefficient) AND variation (e.g. standard deviation) or associated estimates of uncertainty (e.g. confidence intervals) |
| ☒ | ☐ | For null hypothesis testing, the test statistic (e.g. $F$, $t$, $r$) with confidence intervals, effect sizes, degrees of freedom and $P$ value noted<br>*Give P values as exact values whenever suitable.* |
| ☒ | ☐ | For Bayesian analysis, information on the choice of priors and Markov chain Monte Carlo settings |
| ☒ | ☐ | For hierarchical and complex designs, identification of the appropriate level for tests and full reporting of outcomes |
| ☒ | ☐ | Estimates of effect sizes (e.g. Cohen's $d$, Pearson's $r$), indicating how they were calculated |

*Our web collection on statistics for biologists contains articles on many of the points above.*

## Software and code

Policy information about availability of computer code

| | |
|---|---|
| Data collection | Extraction software G2567AA version A.11.5.1.1 (Agilent Technologies)<br>Rosetta Resolver Biosoftware, build 7.2.2 SP1.31 (Rosetta Biosoftware)<br>LAS X (Leica Microsystems) |
| Data analysis | fgsea R package (bioconductor)<br>R version 3.3<br>Graph Pad Prism 7 Software<br>Microsoft Excel<br>Image J/ Fiji<br>StepOne™ Real-Time PCR Software v2.2 (Applied Biosytems) |

For manuscripts utilizing custom algorithms or software that are central to the research but not yet described in published literature, software must be made available to editors/reviewers. We strongly encourage code deposition in a community repository (e.g. GitHub). See the Nature Research guidelines for submitting code & software for further information.

## Data

Policy information about availability of data

All manuscripts must include a data availability statement. This statement should provide the following information, where applicable:

- Accession codes, unique identifiers, or web links for publicly available datasets
- A list of figures that have associated raw data
- A description of any restrictions on data availability

The microarray data from this manuscript have been deposited in the National Centre for Biotechnology Information Omnibus (GEO) under accession code GSE115752.

Quantitative data supporting the findings of this study are available within the paper and its supplementary information files. All other data supporting these findings are available from corresponding author upon reasonable request.
The source data underlying Figs. 1b,c,h,i 2a,b,c,f,h, 3a,b,f,h,j, 4b,d,f,h, 5b,g, 6c,d,e,g, 7c,e,g and Supplementary Figs. S2b, S3a, S5a,b are provided in the Source Data File.

# Field-specific reporting

Please select the one below that is the best fit for your research. If you are not sure, read the appropriate sections before making your selection.

☒ Life sciences ☐ Behavioural & social sciences ☐ Ecological, evolutionary & environmental sciences

For a reference copy of the document with all sections, see nature.com/documents/nr-reporting-summary-flat.pdf

# Life sciences study design

All studies must disclose on these points even when the disclosure is negative.

**Sample size**
No statistical methods were used to predetermine sample size. The samples size was based on availabilit yof mice with the respective genotype and previous experience

**Data exclusions**
No data were excluded from analysis

**Replication**
All attempts at replication were successful. Graphs represent data with at least three biological replicates, all images represent findings reproduced at least twice in the laboraotory

**Randomization**
Animal experiments were done on littermates randomly allocated to different experimental groups.

**Blinding**
The investigator was blinded for analysis of the data (microarray, qpcr, image anaysis)

# Reporting for specific materials, systems and methods

We require information from authors about some types of materials, experimental systems and methods used in many studies. Here, indicate whether each material, system or method listed is relevant to your study. If you are not sure if a list item applies to your research, read the appropriate section before selecting a response.

## Materials & experimental systems

| n/a | Involved in the study | |
|---|---|---|
| ☐ | ☒ | Antibodies |
| ☒ | ☐ | Eukaryotic cell lines |
| ☒ | ☐ | Palaeontology |
| ☐ | ☒ | Animals and other organisms |
| ☒ | ☐ | Human research participants |
| ☒ | ☐ | Clinical data |

## Methods

| n/a | Involved in the study | |
|---|---|---|
| ☒ | ☐ | ChIP-seq |
| ☒ | ☐ | Flow cytometry |
| ☒ | ☐ | MRI-based neuroimaging |

## Antibodies

**Antibodies used**
mouse-anti-e-cadherin (BD Pharmingen ,Cat.:610181, Lot: 6315829, 1:300)
rabbit-anti-Aquaporin 8 (Bioss, Cat.: Bs-6786R, Lot:AC01171331, 1:100)
rat-anti-Ki67 (ThermoFisher, Cat 11-5698-82, Lot:4311665, 1:100)
rabbit- anti-Ki67 (Cell Signaling, Cat.:9192S, Lot:3, 1:100)
rabbit-anti-Krt20 (Cell Signaling, Cat.: 13063, Lot: 1 1:200)
DAPI (Roche, Cat.: 10236276001. Lot:70474820, 1:300)
mouse-anti-EpCAM (Miltenyi Biotech, Cat.:130-102-234, Lot:5161201178, 1:10)
donkey-anti-mouse Alexa Flour647 (Dianova, Cat.:715-605-150, Lot:109506, 1:250)
donkey-anti-rabbit Alexa Flour488 (Dianova, Cat.:711-546-152, Lot:129486, 1:250)
donkey-anti-rabbit Cy3 (Dianova, Cat.:711-165-152, Lot:117211, 1:250)
Phalloidin AlexaFlour647 (Invitrogen, Cat.:A22287, Lot: 1731699, 1:100)

**Validation**
For the antibidies used, there was already a known pattern of protein expression in situ. The localization within the cell (e.g nucvear versus cytoplasmatic) and within the tissue (e.g. base or surface of the crypt) was investigated for each antibody at the expected pattern was confirmed. In addition, single cell RNAseq data (Tabula muris) from the colon were used to confirm the expression in a distinct cell type. Secondary antibody only controls were used. Moreover, validation experiments for the respective antibodies were performed by the commercial vendor and we provide the respective link for each antibody below:

mouse-anti-e-cadherin
http://www.bdbiosciences.com/ds/pm/tds/610181.pdf
rabbit-anti-Aquaporin 8
https://www.biossusa.com/products/bs-6786r
rat-anti-Ki67 :
https://www.thermofisher.com/antibody/product/Ki-67-Antibody-clone-SolA15-Monoclonal/11-5698-82
rabbit- anti-Ki67
https://www.cellsignal.de/products/primary-antibodies/ki-67-d3b5-rabbit-mab/9129
rabbit-anti-Krt20
https://www.cellsignal.de/products/primary-antibodies/keratin-20-d9z1z-xp-rabbit-mab/13063 (IF)
mouse-anti-EpCAM
https://www.miltenyibiotec.com/DE-en/products/macs-flow-cytometry/antibodies/primary-antibodies/cd326-epcam-antibodies-mouse-caa7-9g8-1-10.html#apc:30-ug-in-1-ml
Phalloidin AlexaFlour647
https://www.thermofisher.com/order/catalog/product/A22287

## Animals and other organisms

Policy information about studies involving animals; ARRIVE guidelines recommended for reporting animal research

| | |
|---|---|
| Laboratory animals | For the study 6-8 week old male mice were used. The following genetic strains were used:<br>Axin2CreErt2/Rosa26-tdTomato<br>Lgr5DTReGFP<br>Myh11CreErt2/Rspo3fl/fl<br>Myh11CreErt2/Rspo3+/+<br>Krt20CreErt2/Rosa26-tdTomato<br>Lgr5CreErt2/Rosa26-tdTomato<br>Lgr5DTR/Myh11CreErt2/Rspo3fl/fl<br>Lgr5DTR/Axin2CreErt2/Rosa26-tdTomato |
| Wild animals | Study did not involve wild animals |
| Field-collected samples | No field collected samples were used |
| Ethics oversight | Animal studies were approved by the local authorities (LaGeSo Berlin) |

Note that full information on the approval of the study protocol must also be provided in the manuscript.
